# Tailored Porous Bimetallic Nanozyme Platform for Full‐Cycle Therapeutics of Intestinal Ischemia/Reperfusion

**DOI:** 10.1002/advs.202520748

**Published:** 2026-03-31

**Authors:** Chenghao Qiu, Wenjing Xian, Mengxue Wang, Guozhi Zhao, Yanhuo Zhang, Zhiyu Zhou, Bo Sheng, Zhuo Zhen, Xie Liu, Shouwen Diao, Mengwei Tu, Chen Qiu, Huixian Wang, Jianbo Zhang, Peng Zhu, Tong Li

**Affiliations:** ^1^ Department of Gastrointestinal Surgery The Second Affiliated Hospital of Chongqing Medical University Chongqing P. R. China; ^2^ Department of Anesthesiology The First Affiliated Hospital of Chongqing Medical University Chongqing P. R. China; ^3^ Department of Urology Surgery The First Affiliated Hospital of Chongqing Medical University Chongqing P. R. China; ^4^ Department of Respiratory Medicine The Second Affiliated Hospital of Chongqing Medical University Chongqing P. R. China; ^5^ Department of Rehabilitation Medicine The Second Affiliated Hospital of Chongqing Medical University Chongqing P. R. China; ^6^ Department of Oncology Laboratory of Immunity Inflammation & Cancer The First Affiliated Hospital of Chongqing Medical University Chongqing P. R. China

**Keywords:** antioxidant, dual‐phase therapy, intestinal ischemia/reperfusion injury, Prussian blue nanozyme, tissue repair

## Abstract

Intestinal ischemia/reperfusion (I/R) injury presents a biphasic pathology: an acute oxidative‐inflammatory phase leading to organ failure, and a recovery phase marked by mucosal dysfunction and bacterial translocation. The developed MPB@TA‐Cu‐Ma nanocomposite functions as a dual‐phase therapeutic platform with significant efficacy. It rapidly scavenges reactive oxygen species (ROS) (exhibiting a 50.15% higher clearance in vitro) and suppresses macrophage pyroptosis within 6 h post‐I/R. Furthermore, it enhances mucosal integrity (1.98‐fold Occludin upregulation) and angiogenesis (3.6‐fold increase in CD31^+^ cells) by 96 h. Transcriptomic and immunohistochemical analyses identify three key mechanisms underlying this efficacy: (1) inhibition of the NOD‐like receptor family pyrin domain‐containing 3 (NLRP3)/ cysteinyl aspartate‐specific proteinase 1 (caspase‐1) pathway to suppress pyroptosis; (2) upregulation of defensin alpha 1 (DEFA1) and desmoglein 1 (DSG1) for epithelial repair; and (3) enhancement of vascular endothelial growth factor (VEGF) and angiotensin‐converting enzyme (ACE) expression for vascular regeneration. Overall, MPB@TA‐Cu‐Ma achieves synchronized, phase‐adaptive therapy by disrupting the oxidative‐inflammatory‐barrier axis through enhanced ROS scavenging, enzyme‐mimetic activity, promotion of angiogenesis, and immune modulation, thereby effectively addressing the complex biphasic pathology of intestinal I/R injury.

## Graphical abstract

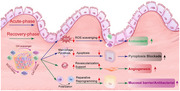

## 1 Introduction

Intestinal ischemia/reperfusion (I/R) injury presents a critical clinical challenge characterized by a biphasic pathological progression. The acute phase (0–24 h post‐injury) features mutually reinforcing oxidative stress and systemic inflammatory cascades that frequently progress to septic shock and multi‐organ dysfunction syndrome (MODS) [1, 2, 3]. In the recovery phase (>72 h), compromised intestinal barrier function predisposes patients to gut microbiota translocation and secondary sepsis [4, 5]. Current therapeutic interventions demonstrate limited efficacy due to an inability to address the spatiotemporal complexity of I/R pathophysiology [6]. An optimal intervention should combine acute‐phase reactive oxygen species (ROS) scavenging and inflammation control with recovery‐phase inflammation resolution and tissue regeneration [7, 8].

To address acute‐phase oxidative damage, Prussian blue (PB) nanoparticles have emerged as a rationally designed therapeutic platform. As the only U.S. Food and Drug Administration (FDA)‐approved antioxidant nanozyme [9], PB nanoparticles demonstrate structural robustness, exceptional biocompatibility, and tunable multi‐enzymatic activities, simultaneously mimicking superoxide dismutase (SOD) and catalase (CAT) functions [10]. However, conventional PB formulations face three critical limitations: suboptimal antioxidant capacity against complex oxidative stress microenvironments, an inability to promote tissue regeneration, and restricted drug‐loading efficiency that hinders combination therapies [11].

The pathophysiological progression of I/R injury necessitates phase‐specific modulation of macrophage functionality. During the acute phase, macrophage pyroptosis acts as a critical amplifier of inflammatory cascades [12], where ROS‐mediated mitochondrial damage triggers gasdermin D‐dependent pore formation and cytokine release [13, 14]. In the recovery phase, macrophage M1‐to‐M2 phenotypic switching becomes pivotal for inflammation resolution through interleukin‐10 (IL‐10) and transforming growth factor‐beta (TGF‐β) secretion, as well as efferocytosis‐mediated debris clearance [15, 16, 17]. Meanwhile, M2‐polarized macrophages facilitate tissue repair via matrix remodeling [18]. This dual‐phase macrophage regulation highlights the therapeutic potential of coordinating both inflammatory suppression and repair promotion.

Emerging evidence suggests that maresin 1 (MaR1), a specialized pro‐resolving mediator (SPM)[19, 20], may fulfill this dual regulatory role through phase‐dependent mechanisms. Preclinical studies indicate that MaR1 can suppress acute‐phase pyroptosis by inhibiting cysteinyl aspartate‐specific proteinase 1 (caspase‐1) activation and preserving mitochondrial integrity [21], while potentially promoting recovery‐phase M2 polarization via retinoic acid receptor‐related orphan receptor alpha (RORα)‐mediated transcriptional reprogramming [22, 23]. However, despite these immunomodulatory properties, MaR1 alone shows limited efficacy in epithelial regeneration, underscoring the need for complementary strategies to enhance mucosal repair [24].

To bridge these therapeutic gaps, we engineered a phase‐adaptive nanoplatform (MPB@TA‐Cu‐Ma) through strategic PB modification [25]. Tannic acid (TA) etching enhanced drug‐loading capacity while improving ROS scavenging performance [26, 27]. Furthermore, the partial substitution of Fe^3^^+^ with Cu^2^^+^ in the PB lattice achieved dual optimization: (1) synergistic SOD‐ and CAT‐mimic activities for enhanced ROS elimination [28], and (2) pro‐angiogenic capacity to stimulate mucosal repair [29, 30, 31]. Finally, MaR1 encapsulation enabled temporal control of macrophage responses—inhibiting pyroptosis during acute injury while promoting M2 polarization during recovery [32, 33, 34].

**SCHEME 1:**
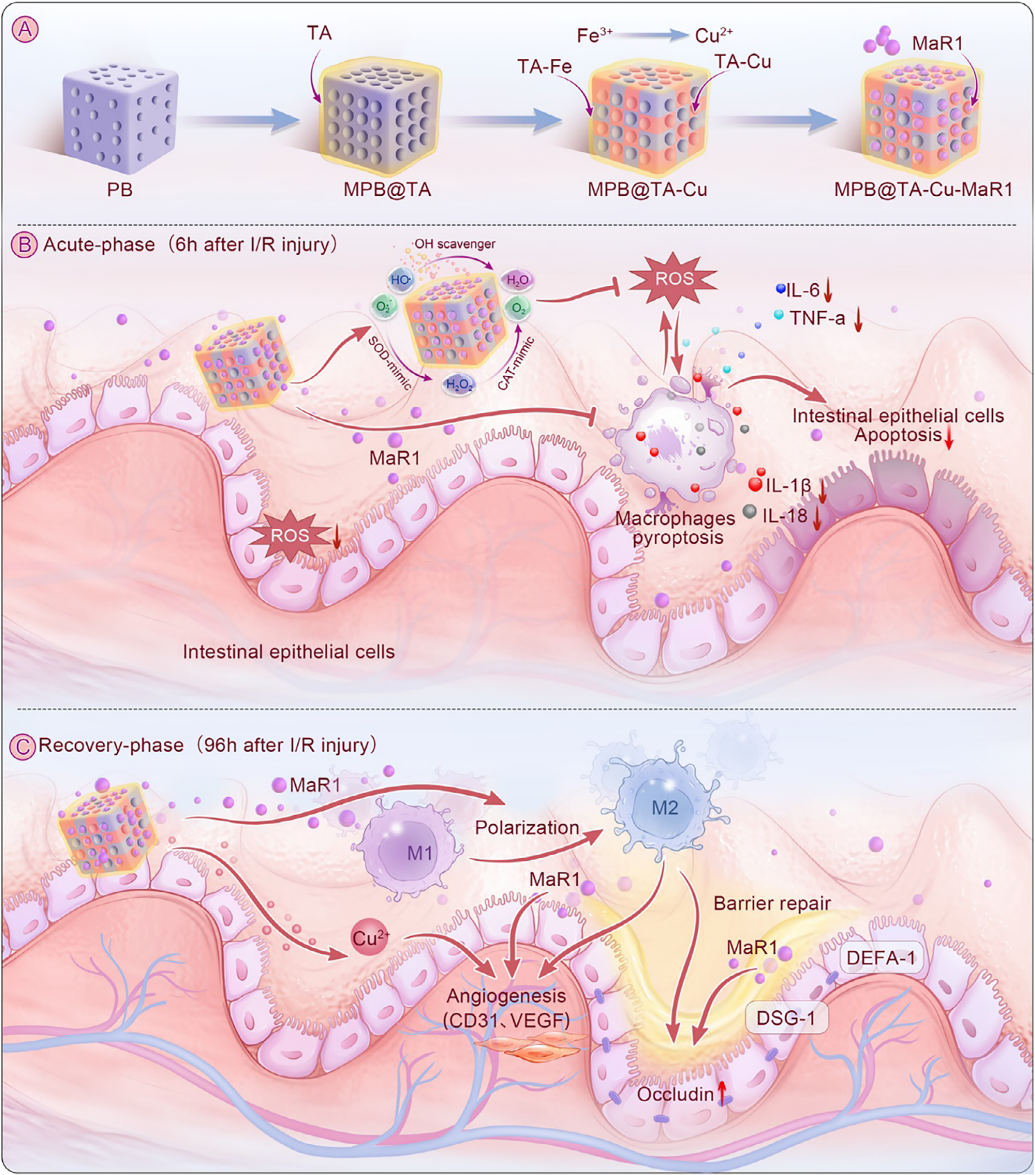
(A) Schematic illustration of the synthetic process and therapeutic mechanisms in the acute phase (B) and recovery phase (C) of MPB@TA‐Cu‐Ma.

In this study, a series of experiments demonstrated that this multifunctional platform effectively scavenged ROS (exhibiting a 50.15% reduction following MPB@TA‐Cu‐Ma treatment in vitro) and suppressed pyroptosis during acute intestinal I/R injury. At 96 h post‐injury, it enhanced mucosal integrity (1.98‐fold increase in Occludin expression) and stimulated angiogenesis (3.6‐fold increase in CD31 expression). Transcriptomic studies elucidated the molecular mechanisms underlying these observed therapeutic effects (Scheme 1).

## 2 Results

### 2.1 Structural and Compositional Characterization of PB, MPB@TA, and MPB@TA‐Cu

**FIGURE 1:**
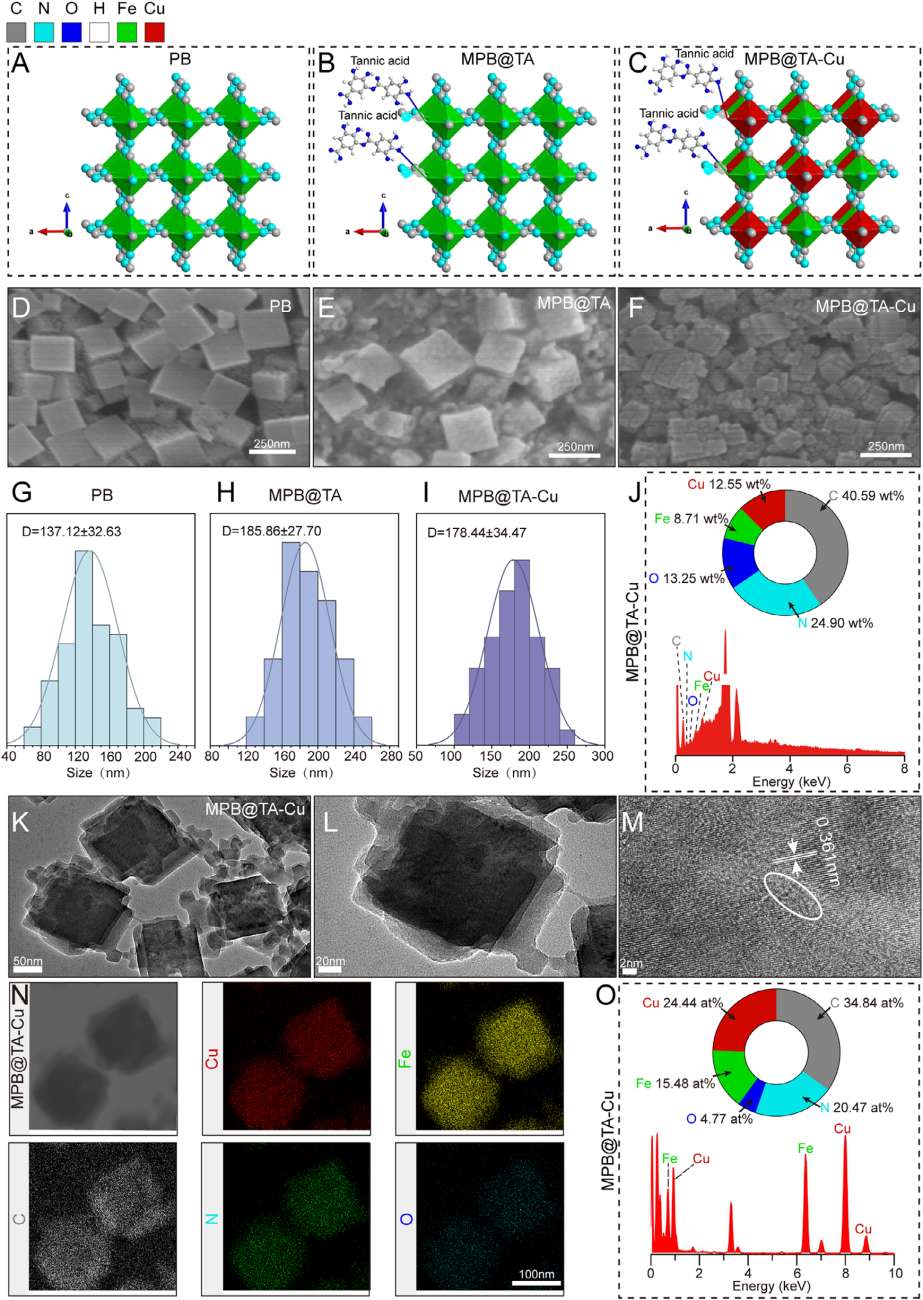
(A–C) Schematic models of PB, MPB@TA, and MPB@TA‐Cu. (D–F) SEM images showing surface morphology of PB, MPB@TA, and MPB@TA‐Cu. (G–I) Particle size distribution histograms of PB, MPB@TA, and MPB@TA‐Cu. (J) XPS spectrum and elemental composition of MPB@TA‐Cu from SEM analysis. (K–M) TEM images of MPB@TA‐Cu at varying magnifications. (N) TEM‐based elemental mapping of MPB@TA‐Cu. (O) XPS spectrum and elemental composition of MPB@TA‐Cu from TEM analysis.

Structural optimization and functionalization of Prussian blue (PB) were achieved through a stepwise modification strategy (Figure 1A–C). First, the selective etching of PB with tannic acid (TA) under acidic conditions generated a mesoporous structure (MPB@TA). Subsequent cation exchange partially substituted Fe^3^^+^ with Cu^2^^+^, yielding the final composite, MPB@TA‐Cu.

Scanning electron microscopy (SEM) images revealed that pristine PB exhibited a smooth cubic morphology with an average particle size of 137.12 nm (Figure 1D, G). TA etching induced significant surface roughening in MPB@TA, which was attributed to the preferential cleavage of Fe^3^^+^─N≡C bonds by TA. This process simultaneously generated a mesoporous structure and formed a TA polymer coating rich in catechol groups, achieved through coordination between the catechol groups of TA and the exposed Fe^3^^+^ ions (Figure 1E) [35]. The surface coordination by TA further induced particle expansion, increasing the average size to 185.86 nm (Figure 1H). Subsequent Cu^2^^+^ exchange reduced the size of MPB@TA‐Cu to 178.44 nm (Figure 1F,I), indicating a lattice contraction caused by the occupation of Fe^3^^+^ vacancies by Cu^2^^+^ ions [36]. SEM coupled with energy‐dispersive X‐ray spectroscopy (SEM‐EDS) analysis revealed a significant reduction in Fe content by 9.38 wt% (from 18.09 wt% in MPB@TA to 8.71 wt% in MPB@TA‐Cu), accompanied by an increase in Cu content to 12.55 wt% (Figure 1J, Figure S1).

Transmission electron microscopy (TEM) further validated the structural uniformity and porosity evolution. Both MPB@TA and MPB@TA‐Cu retained cubic morphologies (Figure 1K and Figure S2), with MPB@TA‐Cu displaying well‐defined mesopores (0.361 nm lattice spacing, Figure 1L, M) characterized by sharp edges and a natural distribution, thereby enhancing the surface area for potential applications. To quantitatively corroborate this visual evidence of enhanced porosity, nitrogen (N_2_) adsorption‐desorption analysis was performed (Figure S3A–C and Table S1). All materials displayed Type I/IV hybrid isotherms with distinct hysteresis loops. Notably, the final MPB@TA‐Cu retained an exceptionally high Brunauer–Emmett–Teller (BET) surface area of 348.46 m^2^/g, while its total pore volume and average Barrett‐Joyner‐Halenda (BJH) pore size expanded to 0.175 cm^3^/g and 3.14 nm, respectively. These results confirmed a robust hierarchical micro/mesoporous network that physically underpinned the subsequent high‐capacity drug loading. Concurrently, regarding the compositional evolution, elemental analysis revealed a significantly higher Fe content in MPB@TA compared to MPB@TA‐Cu, with a distinct Cu signal detected only in MPB@TA‐Cu (Figure 1N). TEM‐EDS analysis of MPB@TA‐Cu also showed a decrease in Fe content (from 22.16 at% to 15.48 at%) (Figure S4) and an increase in Cu content compared with MPB@TA (Figure 1O).

To evaluate the structural stability of MPB@TA‐Cu, SEM observations were conducted over a 96 h incubation period in a physiological buffer (Figure S5). At 0 and 48 h, the nanoparticles retained their cubic morphology and discrete distribution, demonstrating stability during the early stages. After 96 h, some particles exhibited corner rounding, increased surface roughness, and partial collapse, indicating gradual degradation during the simulated drug release process. However, a significant fraction of the nanoparticles retained their structural integrity, with no severe aggregation observed. These findings suggest that MPB@TA‐Cu maintains its overall framework during prolonged release while undergoing controlled degradation, which supports sustained drug delivery and eventual clearance.

### 2.2 XPS and XRD Analysis of PB, MPB@TA, and MPB@TA‐Cu

**FIGURE 2:**
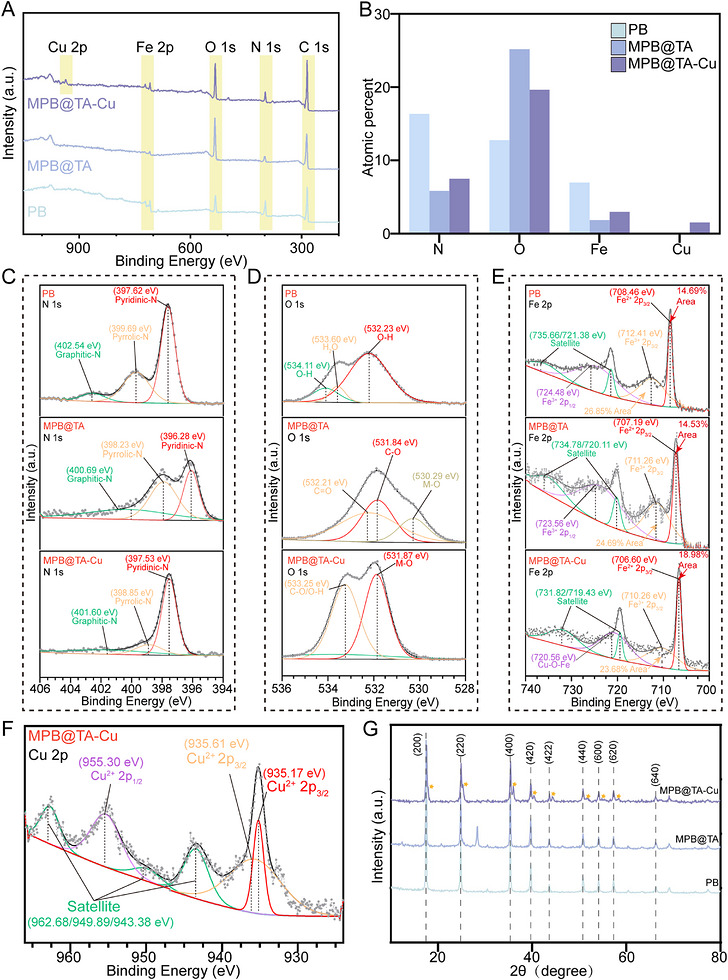
(A) XPS spectra of Cu 2p, Fe 2p, O 1s, N 1s, and C 1s. (B) Atomic percentages (at%) of N, O, Fe, and Cu in the samples. (C–E) High‐resolution XPS spectra of N 1s, O 1s, and Fe 2p for PB, MPB@TA, and MPB@TA‐Cu. (F) High‐resolution Cu 2p spectrum of MPB@TA‐Cu. (G) XRD patterns of PB, MPB@TA, and MPB@TA‐Cu.

X‐ray photoelectron spectroscopy (XPS) full‐spectrum analysis confirmed the stable presence of C, N, O, and Fe in PB, MPB@TA, and MPB@TA‐Cu (Figure 2A), and the surface atomic percentage (at%) of Cu was 1.54 in MPB@TA‐Cu (Figure 2B).

The high‐resolution N 1s spectra revealed systematic shifts in nitrogen configurations following TA modification and Cu^2^^+^ substitution (Figure 2C). For pristine PB, peaks corresponding to pyridinic‐N, pyrrolic‐N, and graphitic‐N were located at 397.62, 399.69, and 402.54 eV, respectively. Following TA modification (MPB@TA), these peaks shifted to 396.28, 398.23, and 400.69 eV. Subsequent Cu^2^^+^ treatment (MPB@TA‐Cu) induced further pronounced changes: the pyridinic‐N peak shifted by +1.25 to 397.53 eV, and the pyrrolic‐N peak shifted by +0.62 to 398.85 eV [37, 38]. Similarly, the high‐resolution C 1s spectra (Figure S6) showed that TA etching eliminated the PB C≡N peak (285.57 eV), and the introduction of Cu^2^^+^ generated a new O─C═O peak at 288.76 eV [39, 40].

The high‐resolution O 1s spectrum of PB exhibited peaks at 532.23 eV (O─H), 533.60 eV (H_2_O), and 534.11 eV (O─H) (Figure 2D). The TA‐modified MPB@TA showed peaks at 531.84 eV (C─O), 532.21 eV (C═O), and 530.29 eV (M─O) [41, 42]. Upon Cu^2^^+^ incorporation into MPB@TA‐Cu, the M–O peak shifted significantly (+1.58 eV) to 531.87 eV, which was attributed to the replacement of Fe^3^^+^ by the more electronegative Cu^2^^+^. Concurrently, the C─O/O─H binding energy shifted to 533.25 eV [43, 44]. These changes indicate Cu^2^^+^‐mediated interactions between the TA ligands and the PB framework, driving surface oxygen redistribution and interfacial structural reorganization [45].

The high‐resolution Fe 2p spectrum of PB (Figure 2E) showed Fe^2^^+^ 2p_3_/_2_ and Fe^3^^+^ 2p_3_/_2_ peaks at 708.46 eV (14.69% area) and 712.41 eV (26.85% area), respectively. Upon TA etching, both peaks exhibited a significant shift to lower binding energies (Fe^2^^+^ 2p_3_/_2_: 707.19 eV, 14.53% area; Fe^3^^+^ 2p_3_/_2_: 711.26 eV, 24.69% area). Subsequent Cu^2^^+^ incorporation led to a further reduction in binding energy (Fe^2^^+^ 2p_3_/_2_: 706.60 eV, 18.98% area; Fe^3^^+^ 2p_3_/_2_: 710.26 eV, 23.68% area). The concurrent decrease in peak intensity (from 24.69% to 23.68% in area for Fe^3^^+^) supported the partial substitution of Fe^3^^+^ by Cu^2^^+^ during structural modification. Furthermore, the MPB@TA‐Cu spectrum revealed a new peak at 720.56 eV (comprising 31.92% of the total spectral area), suggesting the formation of Cu─O─Fe linkages. Combined with the distinct doublet observed in the Cu 2p_3_/_2_ region, these observations collectively substantiate the presence of heterometallic interactions [41, 46].

The high‐resolution Cu 2p spectrum of MPB@TA‐Cu (Figure 2F) resolved two distinct Cu^2^^+^ 2p_3_/_2_ peaks at 935.17 and 935.61 eV, indicating that Cu^2^^+^ species coexisted in two different chemical environments. Additionally, satellite peaks detected at 962.68, 949.89, and 943.38 eV, along with a spin‐orbit splitting energy (Δ = 19.69 eV) between the Cu^2^^+^ 2p_3_/_2_ peak (935.61 eV) and the Cu^2^^+^ 2p_1_/_2_ peak (955.30 eV), confirmed the characteristic d^9^ electronic configuration of Cu^2^^+^. These results collectively demonstrate that MPB@TA‐Cu was successfully formed through the substitution of Fe^3^^+^ by Cu^2^^+^ ions [47].

X‐ray diffraction (XRD) analysis was performed to investigate the crystallographic characteristics of MPB@TA‐Cu (Figure 2G). The diffraction patterns of PB and MPB@TA displayed characteristic peaks at 17.5°, 24.8°, 35.4°, 39.7°, 43.6°, 50.8°, 54.1°, 57.3°, and 66.3°/66.2°, corresponding to the (200), (220), (400), (420), (422), (440), (600), (620), and (640) planes of PB (JCPDS No. 73‐0687), respectively. After Cu^2^^+^ incorporation, the characteristic peaks of MPB@TA‐Cu exhibited a slight shift toward higher diffraction angles (e.g., the (200) plane shifted from 17.5° to 17.7°, Δθ ≤ 0.2°), indicating lattice contraction (Δd ≈ 0.08 Å) [48, 49]. This shift is attributed to the partial substitution of high‐spin Fe^3^^+^ (ionic radius: 0.785 Å) by smaller Cu^2^^+^ ions (0.73 Å). In addition, several new diffraction peaks appeared at 2θ = 25.2°, 36.0°, 40.4°, 44.3°, 51.4°, 54.8°, and 58.4°, suggesting the formation of a Cu‐containing secondary crystalline phase rather than a simple lattice substitution [50, 51].

### 2.3 ROS Scavenging Properties of MPB@TA‐Cu

**FIGURE 3:**
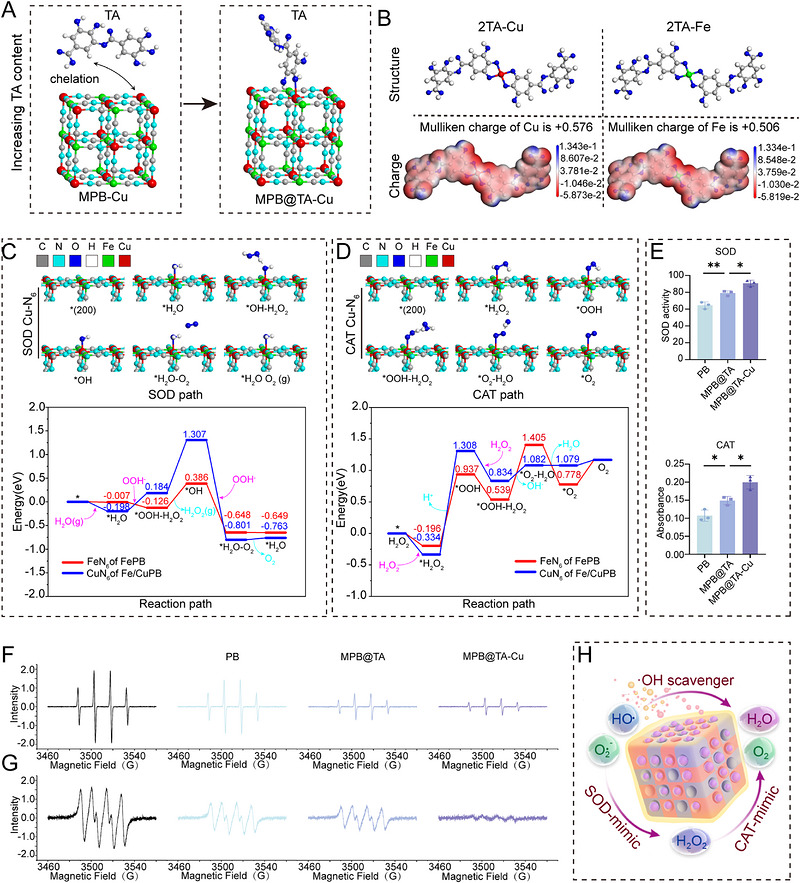
(A) Schematic illustration of TA chelation on MPB‐Cu. (B) Atomic structures and Mulliken charge distribution of 2TA‐Cu and 2TA‐Fe. (C, D) DFT‐calculated intermediate products and reaction barriers for SOD and CAT catalytic pathways with Fe‐N_6_ and Cu‐N_6_ active centers. (E) SOD and CAT activities of PB, MPB@TA, and MPB@TA‐Cu. (F, G) ESR spectra of PB, MPB@TA, and MPB@TA‐Cu for •OH and •O2− scavenging. (H) Schematic of the SOD/CAT enzyme cascade reaction. Data are presented as mean ± SEM (*n* = 3 biologically independent experiments). Statistical analysis was performed using one‐way ANOVA followed by Tukey's post hoc test. *p < 0.05, **p < 0.01; ns, not significant.

Figure 3A and Figure S7 present the computational atomic models of TA adsorption on MPB‐Cu. Surface TA enrichment may enhance catalytic functional group density [52], facilitate interfacial electron transfer, and improve reactive oxygen species (ROS) scavenging efficiency [53]. Mulliken charge analysis (Figure 3B) revealed a higher positive charge on Cu in 2TA‐Cu (+0.576) compared to Fe in 2TA‐Fe (+0.506), which may be attributed to stronger TA‐induced electron withdrawal from Cu^2^^+^ [54].

Density functional theory (DFT) calculations explored the enzymatic mechanisms at the Fe‐N_6_ and Cu‐N_6_ sites using FePB (200) and Fe/CuPB (200) models (Figure 3C,D and Figure S8) [55]. These models clarify the atomic‐level processes underlying superoxide dismutase (SOD)‐mimic and catalase (CAT)‐mimic activities while maintaining structural integrity through charge compensation mechanisms.

For SOD‐mimic activity, given the rapid protonation of •O_2_^−^ in aqueous environments to form OOH• radicals [56], the SOD‐mimic catalytic reaction was simulated by adsorbing OOH• species onto the Fe‐N_6_ and Cu‐N_6_ active sites of the nanozymes. The catalytic cycle began with H_2_O(g) adsorption, forming *H_2_O precursors. Calculated adsorption energies showed a stronger affinity of Cu‐N_6_ for H_2_O(g) (−0.198 eV) compared to Fe‐N_6_ (−0.007 eV), indicating preferential stabilization at the Cu sites. A four‐stage mechanistic progression was analyzed through intermediate configurations, with reaction barriers calculated for each step to assess the impact of active sites on the catalytic pathways (Figure 3C):

(1) ∗H2O+OOH−→∗OH−H2O2

(2) ∗OH−H2O2→∗OH+H2O2

(3) ∗OH+OOH−→∗H2O−O2

(4) ∗H2O−O2→∗H2O+O2(g)

In Step 1, the reaction of adsorbed *H_2_O with OOH^−^ to form *OH–H_2_O_2_ exhibited free energies of −0.119 eV (Fe‐N_6_) and 0.383 eV (Cu‐N_6_). Step 2 involved the decomposition of *OH–H_2_O_2_ to release H_2_O_2_(g) and retain *OH, with free energies of 0.512 eV (Fe‐N_6_) and 1.123 eV (Cu‐N_6_), thermodynamically favoring Fe‐N_6_. Step 3 formed *H_2_O–O_2_ with free energies of −1.034 eV (Fe‐N_6_) and −2.108 eV (Cu‐N_6_), indicating a preference for Cu‐N_6_. In Step 4, *H_2_O–O_2_ decomposed to release O_2_(g) and H_2_O, requiring −0.001 eV (Fe‐N_6_) and 0.038 eV (Cu‐N_6_). The DFT simulations suggest that Cu‐N_6_ facilitates *H_2_O adsorption and *H_2_O–O_2_ formation via OOH^−^ reactions, while Fe‐N_6_ promotes H_2_O_2_(g) release and final O_2_(g) generation. These distinct roles, driven by lower free energy barriers at specific sites, synergistically enhance SOD‐mimic activity.

Figure 3D illustrates the CAT‐mimic intermediate configurations, where H_2_O_2_ (derived from •O_2_^−^ disproportionation) decomposes into H_2_O and O_2_ [57]. The CAT reaction energy profiles showed that the H_2_O_2_ adsorption energy was −0.196 eV (Fe‐N_6_) versus −0.334 eV (Cu‐N_6_), favoring *H_2_O_2_ formation on Cu‐N_6_. The CAT‐mimic mechanism involved five steps (Steps 5–9), with Fe‐N_6_ and Cu‐N_6_ exhibiting divergent adsorption and catalytic behaviors analogous to their SOD‐mimic roles, further rationalizing the site‐specific contributions to cascade reactivity:

(5) ∗H2O2→∗OOH+H++e−

(6) ∗OOH+H2O2(g)→∗OOH−H2O2

(7) ∗OOH−H2O2→∗O2−H2O+OH−

(8) ∗O2−H2O→∗O2+H2O(g)

(9) ∗O2→∗+O2(g)

In Step 5, H_2_O_2_ decomposition to form *OOH required free energies of 1.133 eV (Fe‐N_6_) and 1.642 eV (Cu‐N_6_). Steps 6–7 involved *OOH reacting with H_2_O_2_(g) to form *O_2_–H_2_O and release OH^−^, with free energies of −0.398 eV/0.867 eV (Fe‐N_6_) and −0.474 eV/0.248 eV (Cu‐N_6_), favoring Cu‐N_6_. Step 8 released O_2_(g) and H_2_O(g) via *O_2_–H_2_O decomposition, requiring −0.627 eV (Fe‐N_6_) and −0.003 eV (Cu‐N_6_), indicating an advantage for Fe‐N_6_. Step 9 showed O_2_ desorption energies of 0.389 eV (Fe‐N_6_) and 0.088 eV (Cu‐N_6_), highlighting the efficiency of Cu‐N_6_. The Fe‐N_6_ site appears to drive initial H_2_O_2_ decomposition to *OOH, while Cu‐N_6_ enhances *O_2_–H_2_O formation and O_2_ desorption, facilitating catalytic cycle regeneration.

These calculations suggest that the dual Fe‐N_6_/Cu‐N_6_ sites synergistically reduce the activation energy of the SOD/CAT cascade through differentiated energy barriers and dynamic roles, enabling stepwise ROS scavenging from •O_2_^−^ to H_2_O_2_ and subsequently to H_2_O/O_2_ [58, 59].

Experimentally, the tandem strategy of TA‐mediated etching and Cu^2^^+^ substitution enhanced the in vitro multi‐enzyme‐mimetic performance of MPB@TA‐Cu, as demonstrated by the activity hierarchy (PB < MPB@TA < MPB@TA‐Cu; Figure 3E) [60]. Electron spin resonance (ESR) spectroscopy corroborated this enhancement: (1) TiO_2_/UV‐triggered hydroxyl radicals (•OH) exhibited intensity attenuation (PB > MPB@TA > MPB@TA‐Cu; Figure 3F), and (2) xanthine/xanthine oxidase (XO/XOD)‐generated •O_2_^−^ showed analogous suppression (Figure 3G). These observations underscore the hierarchical ROS elimination efficacy of MPB@TA‐Cu, which is pivotal for therapeutic applications.

Optimization of the structure and ionic configuration of the nanozyme enhances its antioxidant activity. Computational simulations demonstrated that the intrinsic antioxidative effects of TA, combined with the catalytic functionality of Fe^3^^+^/Cu^2^^+^ active sites, synergistically lowered reaction energy barriers, thereby increasing ROS scavenging capacity (Figure 3H) [61].

### 2.4 MPB@TA‐Cu‐Ma Orchestrates Antioxidative/Anti‐Inflammatory Actions Coupled With Pro‐Repair Functions to Achieve Intestinal Protection

**FIGURE 4:**
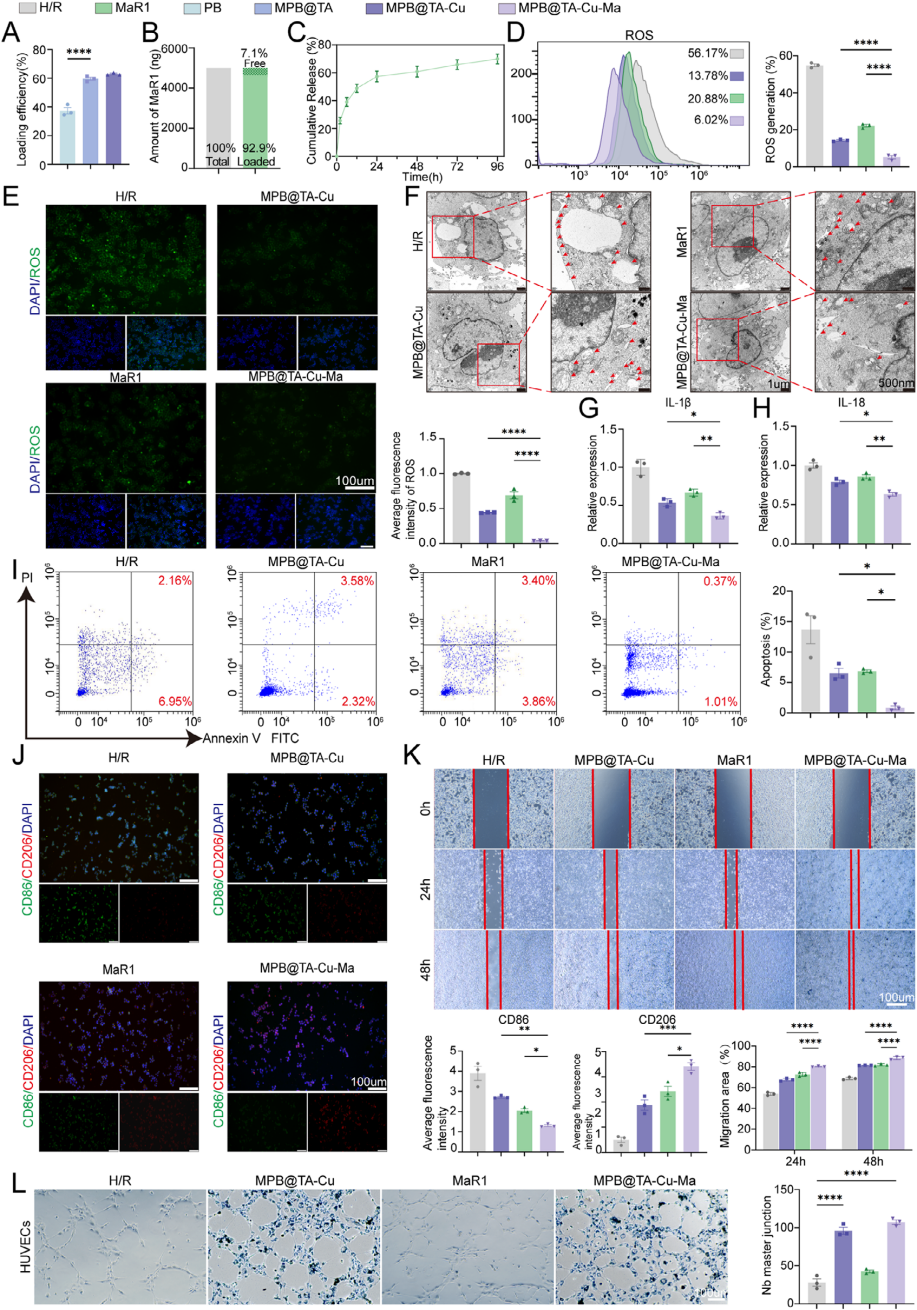
**Characterization of MaR1 loading/release and in vitro therapeutic evaluation of MPB@TA‐Cu‐Ma**. (A) MaR1 loading efficiency (%) in PB, MPB@TA, and MPB@TA‐Cu nanoparticles. (B) Distribution of encapsulated (92.9%) and unencapsulated (7.1%) MaR1 within MPB@TA‐Cu. (C) Cumulative release kinetics of MaR1 from MPB@TA‐Cu in supplemented culture medium at 37°C over 96 h. (D, E) Representative flow cytometry plots (D) and fluorescence images (E) with corresponding quantification showing ROS scavenging in H/R‐treated macrophages after different treatments. Scale bars = 100 µm. (F) Representative TEM images revealing the ultrastructure of macrophages. Red arrows indicate pyroptotic features (e.g., membrane perforation, inflammasomes, and mitochondrial damage). Scale bars = 1 and 500 nm. (G, H) ELISA quantification of pro‐inflammatory cytokines IL‐1β (G) and IL‐18 (H) in macrophage supernatants. (I) Flow cytometric analysis and quantification of apoptosis in MODE‐K cells cultured with macrophage‐derived conditioned medium (CM). (J) Representative immunofluorescence images of macrophage polarization markers (CD86 for M1, green; CD206 for M2, red; DAPI for nuclei, blue) and quantitative fluorescence intensity. Scale bars = 100 µm. (K) Representative images and quantitative migration area of MODE‐K cells in a scratch wound healing assay at 0, 24, and 48 h. Scale bar = 100 µm. (L) Representative images of HUVEC tube formation and quantification of master junctions. Scale bar = 100 µm. Data are presented as mean ± SEM from three biologically independent in vitro experiments (*n* = 3). Statistical significance was determined by one‐way ANOVA (A, G–J, L) and two‐way ANOVA with repeated measures (K) followed by Tukey's post hoc test.

To evaluate the drug delivery capability, we first compared the MaR1 loading efficiencies among the prepared nanoplatforms. The TA‐etched variants (MPB@TA and MPB@TA‐Cu) exhibited a 59.3%–68.2% enhancement compared to unmodified MPB (Figure 4A), validating the necessity of TA‐etching for high‐capacity drug delivery. To further demonstrate the robust payload capacity of the nanoplatform, the system was challenged with a high initial MaR1 input. Subsequent quantitative analysis revealed an exceptional encapsulation profile, with approximately 92.9% of the drug successfully loaded and only 7.1% remaining free (Figure 4B). Following the confirmation of payload capacity, MPB@TA‐Cu exhibited stable and sustained MaR1 release over 96 h in complete culture medium (Figure 4C) and demonstrated a time‐dependent MaR1 release profile in phosphate‐buffered saline (PBS) (Figure S9), alongside the effective concurrent release of copper and iron ions (Figure S10).

Before proceeding to efficacy evaluations, the in vitro biocompatibility of these formulations was confirmed (Figure S11A–D). While unmodified PB slightly suppressed the proliferation of MODE‐K and RAW264.7 cells, all TA‐etched variants maintained high cell viability indistinguishable from the controls. This highlights the crucial role of TA in further optimizing the interfacial biocompatibility of PB, likely through polyphenol‐mediated surface passivation that minimizes non‐specific cellular stress, enhances hydrophilicity, and fosters highly favorable cell‐material interactions.

To evaluate antioxidative efficacy, MODE‐K and RAW264.7 cells were pretreated with nanozymes or MaR1 and then subjected to hypoxia/reoxygenation (H/R) injury. Flow cytometry analyses and fluorescence microscopy in RAW264.7 cells demonstrated that MPB@TA‐Cu‐Ma significantly reduced intracellular ROS levels by approximately 50.15% compared to the H/R controls, exhibiting superior efficacy to both MPB@TA‐Cu (42.39% reduction) and free MaR1 (35.29% reduction) (Figure 4D,E). Similarly, fluorescence microscopy analysis confirmed a marked decrease in ROS levels in the MPB@TA‐Cu‐Ma group relative to the H/R group of MODE‐K cells (Figure S12).

Since macrophage pyroptosis plays a pivotal role in the development of inflammation, we next studied the impact of MPB@TA‐Cu‐Ma on macrophage pyroptosis. The results demonstrated that H/R modeling induced significant cell pyroptosis, as evidenced by increased cell membrane perforation, mitochondrial damage, inflammasome formation, elevated macrophage gasdermin D N‐terminal (GSDMD‐N) expression, and the enhanced release of interleukin‐1 beta (IL‐1β), interleukin‐18 (IL‐18), tumor necrosis factor‐alpha (TNF‐α), and interleukin‐6 (IL‐6). In contrast, treatment with MPB@TA‐Cu‐Ma reduced cell membrane perforation (Figure 4F), mitigated mitochondrial damage and inflammasome formation, decreased macrophage GSDMD‐N expression (Figure S13), and suppressed the release of IL‐1β (Figure 4G), IL‐18 (Figure 4H), TNF‐α, and IL‐6 (Figure S14).

Additionally, we used the culture media (CM) from the aforementioned macrophage groups to cultivate intestinal mucosal epithelial cells. The results showed that the CM from H/R‐treated macrophages induced apoptosis in mucosal epithelial cells, whereas the CM from MPB@TA‐Cu‐Ma‐treated macrophages significantly reduced mucosal cell apoptosis, with an effect superior to that of MaR1 and MPB@TA‐Cu (Figure 4I). These findings indicate that MPB@TA‐Cu‐Ma can mitigate the two key factors that promote the onset and progression of inflammation: ROS overproduction and macrophage pyroptosis.

We then investigated the effects of MPB@TA‐Cu‐Ma on biological functions crucial for tissue repair processes, including macrophage polarization, intestinal epithelial cell migration, and human umbilical vein endothelial cell (HUVEC) tube formation ability. The results revealed that H/R modeling induced the M1 polarization of macrophages, while MPB@TA‐Cu‐Ma promoted the phenotypic transition of macrophages from M1 to M2 (Figure 4J). Furthermore, MPB@TA‐Cu‐Ma enhanced the migration ability of MODE‐K cells (Figure 4K) and promoted the tube formation ability of HUVECs, with these functions being more pronounced than those in the MaR1 and MPB@TA groups (Figure 4L and Figures S15, S16). These in vitro experimental results suggest that MPB@TA‐Cu‐Ma not only scavenges peroxides and controls the inflammatory cascade but also promotes tissue repair and facilitates inflammation resolution.

### 2.5 MPB@TA‐Cu‐Ma Alleviates Intestinal I/R Injury Through Antioxidant, Anti‐Inflammatory, and Pyroptosis‐Inhibition During the Acute‐Phase

To define the optimal therapeutic window, a dose‐escalation study was performed in an intestinal ischemia/reperfusion (I/R) injury model (Figure S17). Mice received MPB@TA‐Cu‐Ma at 0.2, 2, or 4 mg/mL (100 µL; containing 15, 150, and 300 ng MaR1, respectively). Analyses of TNF‐α expression and terminal deoxynucleotidyl transferase dUTP nick end labeling (TUNEL)‐positive apoptotic cells during the acute phase (6 h post‐reperfusion), alongside Occludin levels during the recovery phase (96 h post‐reperfusion), revealed a clear dose‐dependent efficacy. Notably, these therapeutic benefits plateaued at 2 mg/mL, with no further significant improvements observed at the 4 mg/mL dose. Therefore, this optimal 2 mg/mL dose was utilized for all subsequent in vivo evaluations.

We then assessed the biosafety of MPB@TA‐Cu (2 mg/mL) by evaluating copper ion clearance, hepatic and renal functions, and organ histopathology. The nanozyme induced neither long‐term heavy metal accumulation nor functional abnormalities in the liver and kidneys (Figure S18A–F). Furthermore, hematoxylin and eosin (H&E) staining of major organs (heart, liver, spleen, lung, and kidney) at two weeks post‐injection showed no apparent pathological changes across all treatment groups, confirming excellent in vivo biocompatibility (Figure S18G).

**FIGURE 5:**
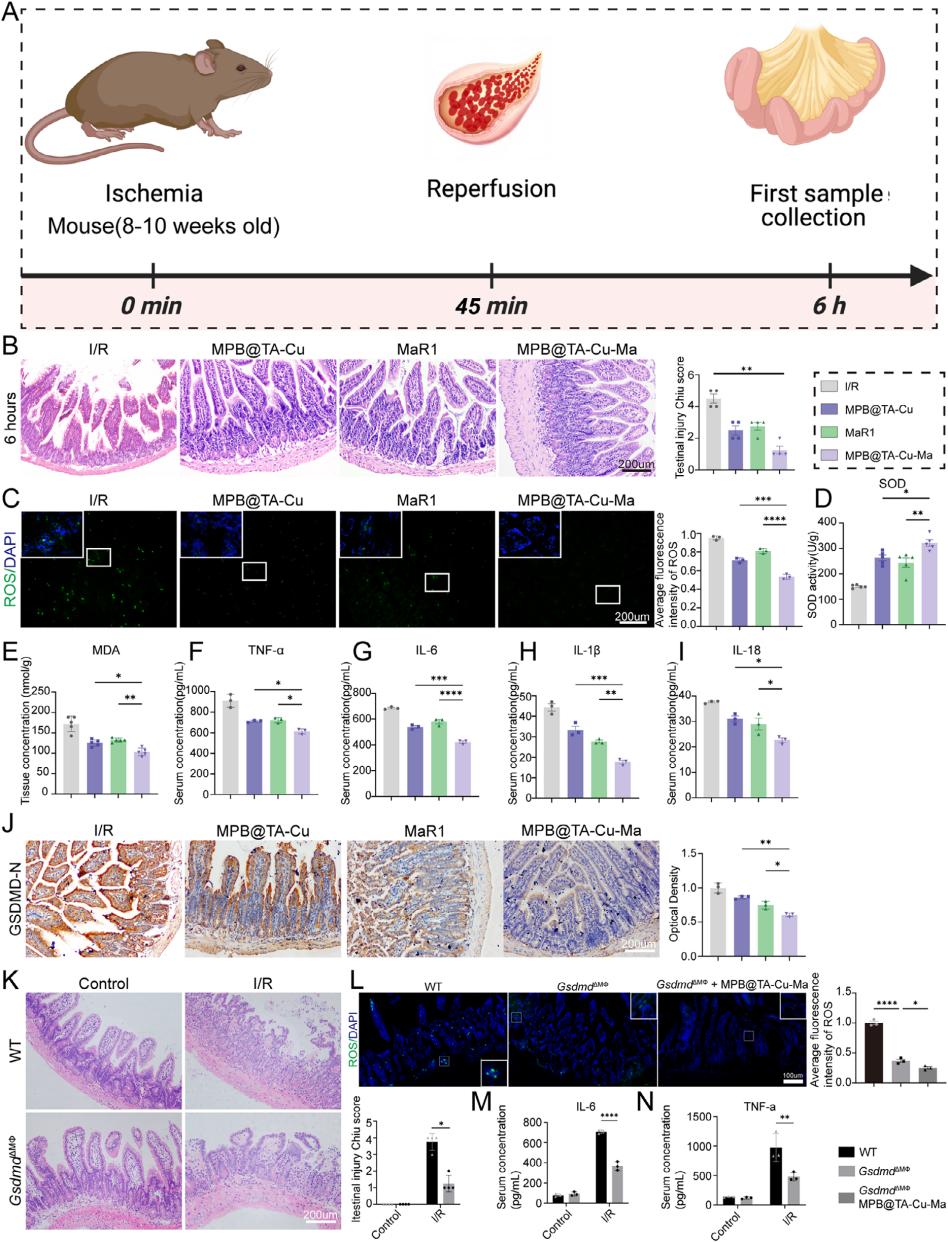
In vivo therapeutic efficacy of MPB@TA‐Cu‐Ma in an intestinal I/R injury model and the mechanism of GSDMD‐mediated pyroptosis. (A) Schematic illustration of the experimental timeline for the intestinal ischemia (45 min) and reperfusion (6 h) model in C57BL/6 mice. (B) Representative H&E‐stained images and histopathological Chiu scores of intestinal tissues from different groups. Scale bar = 200 µm. (C) ROS fluorescence signal (green) in intestinal tissues detected using a fluorescent probe (DCFH‐DA), and quantitative histograms; scale bars: 200 µm. (D, E) Relative SOD activity and MDA concentration in intestinal tissues (*n* = 5 biologically independent mice per group). (F–I) Serum concentrations of pro‐inflammatory cytokines TNF‐α (F), IL‐6 (G), IL‐1β (H), and IL‐18 (I) across different experimental groups as measured by ELISA. (J) Representative immunohistochemistry (IHC) images and quantitative optical density analysis of GSDMD‐N expression in intestinal tissues. Scale bar = 200 µm. (K) HE‐stained small intestinal sections from WT and *Gsdmd* ^ΔMΦ^ mice in Control and I/R groups, with quantitative injury analysis using the Chiu score. Scale bar: 200 µm. (L) ROS fluorescence in frozen small intestinal sections from WT, *Gsdmd* ^ΔMΦ^, and *Gsdmd* ^ΔMΦ^ + MPB@TA‐Cu‐Ma mice after I/R injury, with quantitative analysis of fluorescence intensity. Scale bar: 100 µm. (M‐N) Quantitative analysis of serum IL‐6 and TNF‐α levels in WT and *Gsdmd* ^ΔMΦ^ C57BL/6 mice from Control and I/R groups. Data are presented as mean ± SEM from biologically independent mice (*n* = 4 for B, K;*n* = 5 for D–E; *n* = 3 for all other panels). Statistical analysis was performed using one‐way ANOVA (C–J, L) and two‐way ANOVA (M–N) followed by Tukey's post hoc test. For ordinal data (Chiu scores in B and K), statistical significance was determined using the non‐parametric Kruskal–Wallis test followed by Dunn's multiple comparisons test (B) and the Mann–Whitney U test (K). ****p < 0.0001, ***p < 0.001, **p < 0.01, *p < 0.05; ns, not significant.

We subsequently investigated the temporal protective effects of MPB@TA‐Cu‐Ma on intestinal I/R injury during both the acute phase (6 h post‐reperfusion) and the recovery phase (96 h post‐reperfusion) in C57BL/6 mice. Animals pretreated with MPB@TA‐Cu, MaR1, or MPB@TA‐Cu‐Ma were subjected to small intestinal I/R injury (Figure 5A and Figure S19). At 6 h post‐reperfusion, H&E staining revealed that MPB@TA‐Cu‐Ma markedly alleviated mucosal detachment and intestinal wall edema compared with the I/R group, as evidenced by significantly reduced Chiu scores (Figure 5B). Furthermore, biochemical assessments demonstrated that this treatment effectively reduced ROS accumulation (Figure 5C) and enhanced SOD activity (Figure 5D), while decreasing malondialdehyde (MDA) levels in intestinal tissues (Figure 5E). Enzyme‐linked immunosorbent assay (ELISA) analysis further showed that MPB@TA‐Cu‐Ma significantly suppressed serum TNF‐α (Figure 5F) and IL‐6 concentrations (Figure 5G). Notably, these protective effects against both oxidative damage and inflammation were markedly stronger than those in the MPB@TA‐Cu or MaR1 groups, consistent with the in vitro findings.

Mechanistic investigations revealed that MPB@TA‐Cu‐Ma significantly reduced serum levels of pyroptosis‐associated inflammatory cytokines IL‐1β (Figure 5H) and IL‐18 (Figure 5I), accompanied by downregulated expression of the pyroptosis‐executing protein GSDMD‐N in intestinal tissues (Figure 5J). To validate the role of macrophage pyroptosis in intestinal I/R pathogenesis, we subjected macrophage‐specific *Gsdmd* knockout (*Gsdmd* ^ΔMΦ^) mice to I/R injury (Figure S20). Compared to wild‐type (WT) mice, *Gsdmd* ^ΔMΦ^ mice exhibited significantly reduced intestinal damage and lower Chiu scores (Figure 5K). While *Gsdmd* deficiency alone partially mitigated local oxidative stress, MPB@TA‐Cu‐Ma treatment further suppressed ROS accumulation in *Gsdmd* ^ΔMΦ^ tissues (Figure 5L). Consequently, systemic inflammation was markedly blunted, with significant reductions in serum IL‐6 (Figure 5M) and TNF‐α (Figure 5N). These data confirm that MPB@TA‐Cu‐Ma confers protection primarily by inhibiting macrophage pyroptosis, alongside synergistic benefits from its intrinsic ROS‐scavenging activity.

### 2.6 MPB@TA‐Cu‐Ma Promotes Intestinal I/R Repair Through Macrophage Polarization Modulation, Mucosal Barrier Restoration, and Vascular Regeneration During the Recovery‐Phase

**FIGURE 6:**
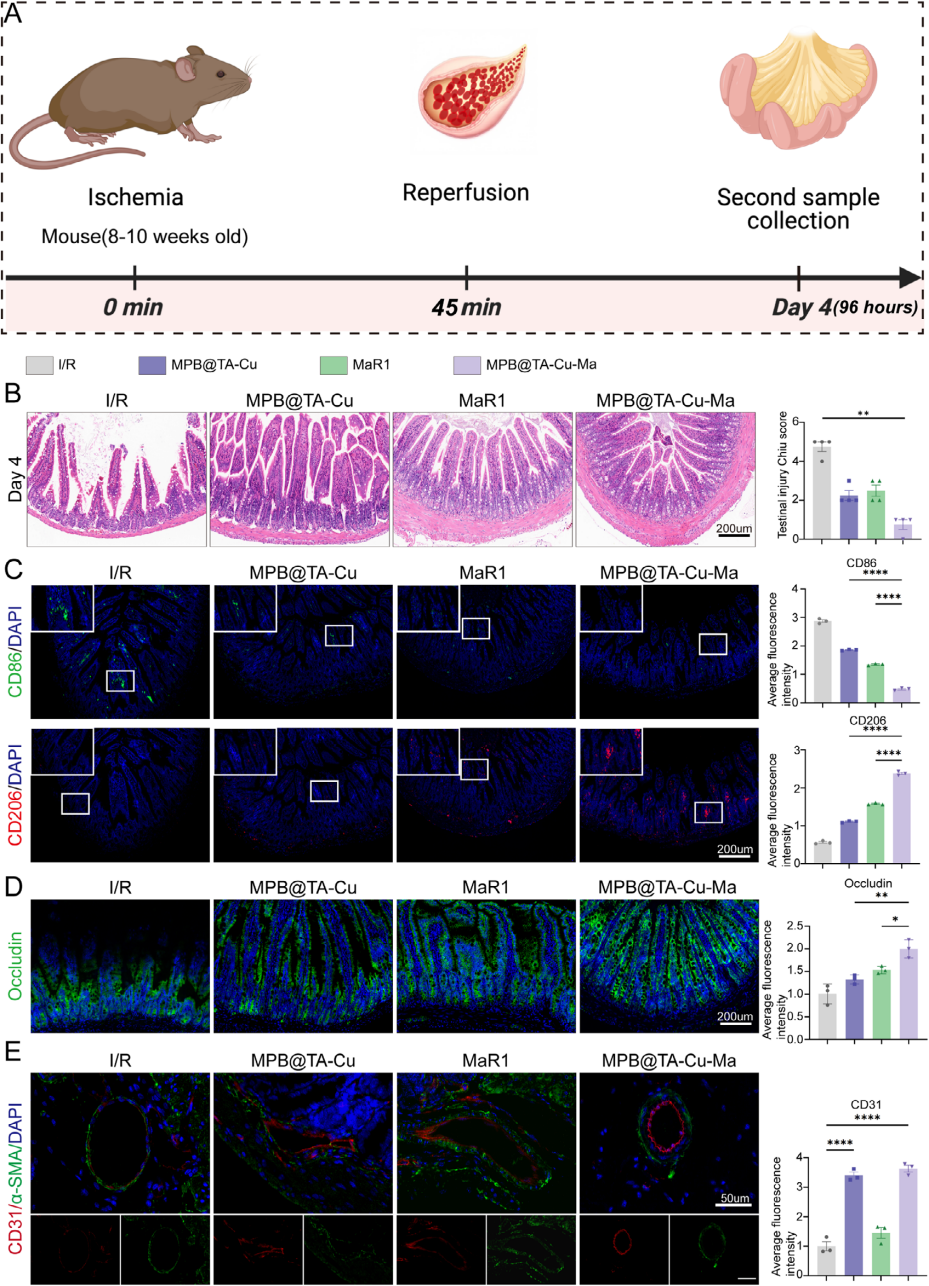
**Therapeutic evaluation of MPB@TA‐Cu‐Ma during the recovery phase of intestinal I/R injury**. (A) Schematic diagram of the ischemia/reperfusion (I/R) model in C57BL/6 mice: 45 min ischemia followed by 4‐day(96‐hour) reperfusion. (B) Representative H&E‐stained images and histopathological Chiu scores of intestinal tissues from different groups at 96 h post‐reperfusion. Scale bar = 200 µm. (C) Representative immunofluorescence images of macrophage polarization markers (M1: CD86, green; M2: CD206, red; DAPI for nuclei, blue) in intestinal tissues and corresponding quantitative analysis of fluorescence intensity. Scale bars = 200 µm. (D) Representative immunofluorescence images and quantitative analysis of the tight junction protein Occludin (green) in intestinal tissues. Scale bar = 200 µm. (E) Representative immunofluorescence images of CD31 (red) and α‐SMA (green) in intestinal tissues, and quantitative analysis of CD31 fluorescence intensity. Scale bars = 50 µm. Data are presented as mean ± SEM from biologically independent mice (*n* = 4 for B; *n* = 3 for all other panels). Statistical significance was determined using one‐way ANOVA followed by Tukey's post hoc test. For ordinal data (Chiu scores in B), the non‐parametric Kruskal–Wallis test followed by Dunn's multiple comparisons test was used. ****p < 0.0001, ***p < 0.001, **p < 0.01, *p < 0.05; ns, not significant.

At 96 h post‐reperfusion (Figure 6A), MPB@TA‐Cu‐Ma demonstrated superior tissue repair capacity compared to the other treatment groups. Histopathological evaluation via H&E staining revealed near‐normal mucosal architecture in the MPB@TA‐Cu‐Ma group. This was characterized by preserved villus integrity, minimal mucosal denudation, and a complete absence of intestinal wall edema (Figure 6B). Consistent with these observations, quantitative assessment using the Chiu scoring system confirmed significantly enhanced histological recovery in this treatment group (Figure 6B).

The reparative mechanism was further elucidated through cellular and molecular analyses. Immunofluorescence profiling demonstrated a remarkable phenotypic shift in macrophage polarization, transitioning from a pro‐inflammatory M1 state to a reparative M2 state (Figure 6C). This immunomodulatory effect was paralleled by the enhanced expression of the tight junction protein Occludin, suggesting the successful restoration of epithelial barrier integrity (Figure 6D). Furthermore, these structural improvements were functionally validated through fluorescein isothiocyanate‐dextran (FD‐4) permeability assays. The results showed significantly reduced intestinal permeability in MPB@TA‐Cu‐Ma‐treated mice compared to the other groups (Figure S21).

Angiogenic potential was confirmed via CD31 immunostaining, revealing a marked upregulation of this vascular endothelial marker in both the MPB@TA‐Cu and MPB@TA‐Cu‐Ma groups. Notably, this enhanced CD31 expression aligned with our previous in vitro tube formation assay results (Figure 6E), establishing strong consistency between the experimental models. While both formulations exhibited therapeutic benefits, the pro‐repair effects across all evaluation parameters were consistently more pronounced in the MPB@TA‐Cu‐Ma group.

To evaluate non‐pyroptosis‐dependent regenerative effects during the recovery phase, we utilized I/R‐challenged *Gsdmd* ^ΔMΦ^mice (Figure S22). While *Gsdmd* deficiency alone partially facilitated mucosal barrier restoration and neovascularization compared to WT mice, the administration of MPB@TA‐Cu‐Ma to *Gsdmd* ^ΔMΦ^ mice induced further significant improvements. Specifically, MPB@TA‐Cu‐Ma‐treated *Gsdmd* ^ΔMΦ^ mice exhibited markedly amplified expression of both Occludin (Figure S22A) and CD31 (Figure S22B) relative to the untreated *Gsdmd* ^ΔMΦ^ group. These data validate that the pro‐repair efficacy of MPB@TA‐Cu‐Ma extends beyond macrophage pyroptosis inhibition, confirming its synergistic capacity to directly stimulate epithelial barrier restoration and angiogenesis.

Collectively, these findings delineate a dual‐phase therapeutic mechanism for MPB@TA‐Cu‐Ma. In the acute phase, it provides robust tissue protection by scavenging ROS and suppressing macrophage pyroptosis to control inflammatory cascades. This is followed by a recovery phase, where it actively drives tissue regeneration through macrophage phenotype switching, mucosal barrier reconstitution, and angiogenic stimulation. The spatiotemporal specificity of these effects, combined with its demonstrated biosafety, positions MPB@TA‐Cu‐Ma as a highly promising therapeutic candidate for the management of I/R injury.

### 2.7 The Molecular Mechanisms by Which MPB@TA‐Cu‐Ma Exerts Tissue‐Protective Functions

**FIGURE 7:**
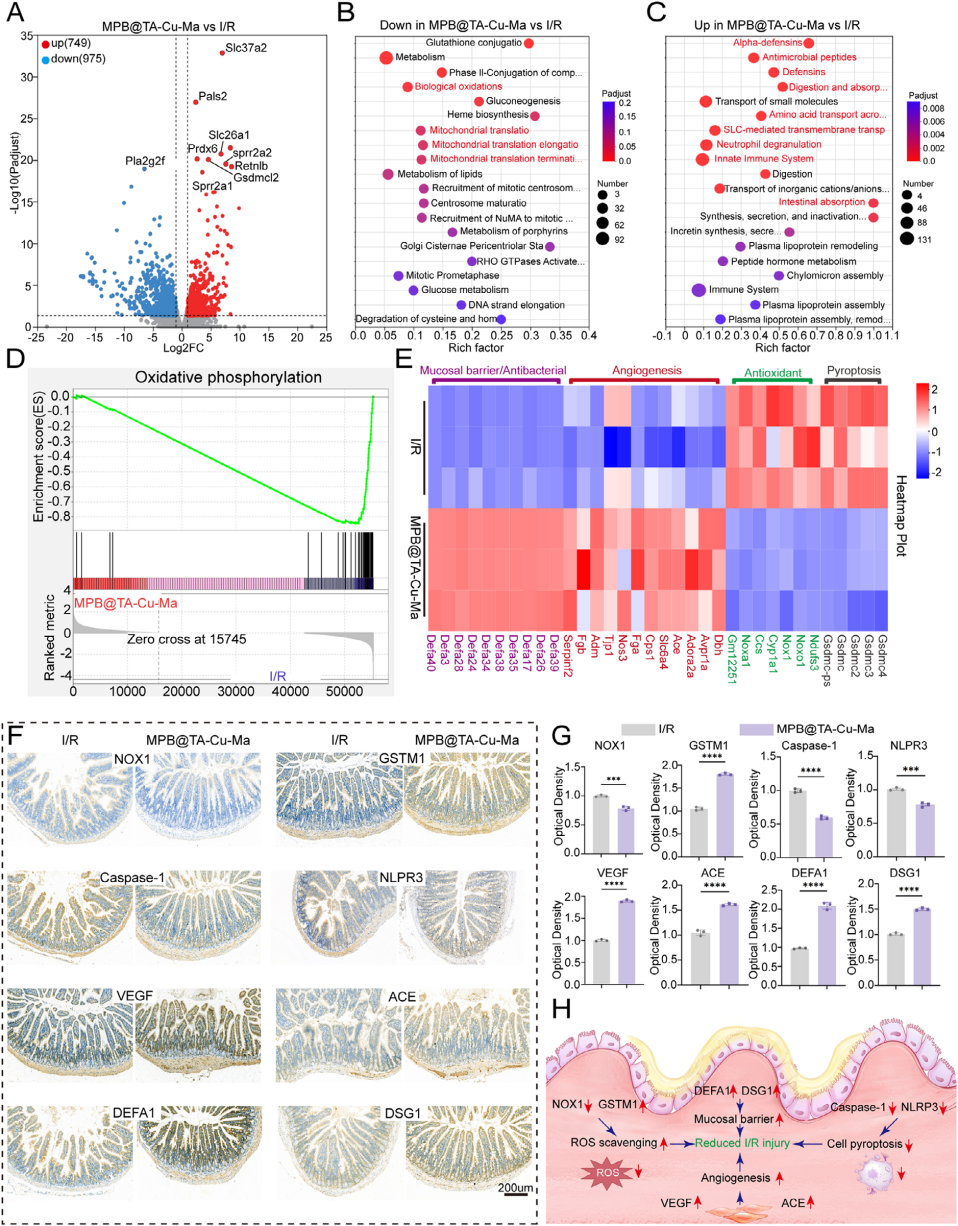
**Transcriptomic analysis and proposed molecular mechanisms underlying the therapeutic effects of MPB@TA‐Cu‐Ma**. (A) Volcano plot of differentially expressed genes (DEGs) comparing the MPB@TA‐Cu‐Ma treatment group with the I/R group. (B, C) Reactome pathway enrichment analysis of downregulated (B) and upregulated (C) genes in the MPB@TA‐Cu‐Ma group versus the I/R group. (D) Gene set enrichment analysis (GSEA) plot highlighting the oxidative phosphorylation pathway. (E) Heatmap of representative DEGs associated with mucosal barrier/antibacterial function, angiogenesis, antioxidant responses, and pyroptosis. (F, G) Representative immunohistochemical (IHC) images (F) and corresponding quantitative optical density analysis (G) of NOX1, GSTM1, Caspase‐1, NLRP3, VEGF, ACE, DEFA1, and DSG1 in intestinal tissues. Scale bar = 200 µm. (H) Schematic illustration summarizing the cascade mechanisms of MPB@TA‐Cu‐Ma in mitigating intestinal I/R injury. Data in (G) are presented as mean ± SEM from n = 3 biologically independent mice. Statistical significance was determined using the non‐parametric Mann–Whitney U test. ****p < 0.0001, ***p < 0.001.

Through transcriptomic analysis, we conducted an in‐depth exploration of gene expression changes in the intestinal tissues of mice from the I/R and MPB@TA‐Cu‐Ma treatment groups. By setting the screening criteria of a fold change > 2 or < 0.5 and an adjusted *p*‐value (*P*_adj_) < 0.05, we identified 1724 differentially expressed genes (DEGs), which were visually represented through a volcano plot (Figure 7A).

Furthermore, we employed Reactome pathway enrichment analysis and Gene Set Enrichment Analysis (GSEA) to identify alterations in signaling pathways between the I/R model group and the MPB@TA‐Cu‐Ma treatment group. Reactome enrichment analysis revealed that downregulated DEGs in the MPB@TA‐Cu‐Ma group (vs. I/R group) were primarily enriched in pathways related to peroxide production (Figure 7B). Conversely, upregulated DEGs were significantly enriched in pathways associated with defensins and tissue proliferation (Figure 7C), suggesting that MPB@TA‐Cu‐Ma simultaneously enhances antibacterial defenses and mucosal repair. Consistently, GSEA demonstrated that the oxidative phosphorylation pathway was predominantly enriched in the I/R group, whereas MPB@TA‐Cu‐Ma treatment significantly modulated this pathway (Figure 7D). Since aberrant mitochondrial respiration during the reperfusion phase is closely associated with ROS leakage, this modulation suggests that the treatment may alleviate I/R injury by regulating mitochondrial metabolic states and mitigating oxidative stress.

Next, we generated a heatmap (Figure 7E) to visualize DEGs related to ROS production, pyroptosis, angiogenesis, and mucosal barrier function. The heatmap showed that DEGs associated with ROS production and pyroptosis, such as NADPH oxidase 1 (*Nox1*), NADH:ubiquinone oxidoreductase core subunit S3 (*Ndufs3*), and gasdermin C (*Gsdmc*), were significantly downregulated in the MPB@TA‐Cu‐Ma group, whereas DEGs related to angiogenesis and barrier integrity, such as nitric oxide synthase 3 (*Nos3*) and defensin alpha 3 (*Defa3*), were upregulated. Expression correlation analysis further revealed the interaction networks among these key genes, elucidating the molecular mechanisms by which MPB@TA‐Cu‐Ma protects the intestinal tissue (Figure S23).

We then assessed the expression of proteins related to ROS production, pyroptosis, angiogenesis, and mucosal barrier function in the intestinal tissues. Compared with the I/R group, the MPB@TA‐Cu‐Ma group exhibited decreased levels of NOX1 (implicated in ROS generation), as well as Caspase‐1 and the NOD‐like receptor family pyrin domain‐containing 3 (NLRP3) (the latter two are core mediators of pyroptosis). Conversely, the MPB@TA‐Cu‐Ma group demonstrated increased expression of glutathione S‐transferase mu 1 (GSTM1) (involved in ROS scavenging), vascular endothelial growth factor (VEGF), and angiotensin‐converting enzyme (ACE) (related to angiogenesis), as well as defensin alpha 1 (DEFA1) and desmoglein‐1 (DSG1) (associated with mucosal barrier function) (Figure 7F,G).

In summary, these transcriptomic and protein‐level results suggest that MPB@TA‐Cu‐Ma exerts significant tissue‐protective effects through multiple pathways. A schematic diagram of the relevant molecular mechanisms is presented in Figure 7H.

## 3 Discussion

Intestinal ischemia/reperfusion injury remains a formidable clinical challenge due to its biphasic pathophysiology: acute oxidative‐inflammatory damage within 24 h post‐reperfusion, followed by delayed mucosal barrier dysfunction and bacterial translocation beyond 72 h [4, 6]. Current interventions show limited efficacy, as they fail to address the spatiotemporal complexity of this biphasic progression. Here, we developed a phase‐adaptive nanoplatform, MPB@TA‐Cu‐Ma, rationally engineered to coordinate antioxidative, anti‐pyroptotic, immunomodulatory, and pro‐regenerative responses across both injury phases, overcoming critical limitations of conventional PB formulations [10].

In the acute phase, MPB@TA‐Cu‐Ma exerted robust protection via synergistic ROS scavenging and macrophage pyroptosis inhibition. The bimetallic Fe‐N_6_ and Cu‐N_6_ active sites conferred complementary SOD‐ and CAT‐mimic activities, validated by DFT simulations, ESR spectroscopy, and in vitro assays showing 50.15% intracellular ROS reduction [10, 21, 29]. While the lack of X‐ray absorption spectroscopy (XAS) to directly resolve metal coordination states is a limitation, our DFT models were built on well‐defined crystallographic and compositional data from XRD, XPS, and SEM‐EDS; this computational approach is well‐established in PB analogue research, with prior studies demonstrating strong correlations between DFT predictions and experimental catalytic performance [62]. Functionally, this catalytic synergy disrupted the oxidative stress‐inflammasome positive feedback loop, suppressing the NLRP3/Caspase‐1/GSDMD pathway to reduce IL‐1β and IL‐18 release [13, 14], with genetic validation from macrophage‐specific *Gsdmd* knockout (*Gsdmd* ^ΔMΦ^) mice confirming pyroptosis inhibition as the core acute protective mechanism. Regarding the Fe/Cu ratio, our adopted doping ratio balanced mesoporous framework stability and catalytic activity, achieving homogeneous Cu incorporation with retained structural integrity and high drug loading capacity. As prior work has shown metal stoichiometry modulates bimetallic nanozyme performance [63], future studies will systematically optimize this ratio to maximize catalytic efficacy.

In the recovery phase, MPB@TA‐Cu‐Ma shifted to drive mucosal repair and angiogenesis via two complementary mechanisms. Sustained release of MaR1 promoted macrophage M1‐to‐M2 polarization to resolve inflammation [16, 18], while Cu^2^^+^ release enhanced angiogenic signaling, evidenced by a 3.6‐fold increase in CD31 expression in vivo [29, 47]. The platform also restored intestinal barrier integrity, with a 1.98‐fold upregulation of the tight junction protein Occludin and reduced intestinal permeability. Critically, studies in *Gsdmd* ^ΔMΦ^ mice confirmed that these pro‐regenerative effects are independent of pyroptosis inhibition, demonstrating the platform's dual‐phase functional versatility.

Compared to existing nanotherapeutics for intestinal I/R, MPB@TA‐Cu‐Ma offers distinct translational advantages. Cerium oxide nanoparticles, commonly used antioxidant nanomaterials, rely solely on single redox cycling for ROS scavenging, lacking intrinsic immunomodulatory or pro‐regenerative functions [64]. In contrast, our platform is built on FDA‐approved PB with well‐documented biocompatibility [65]. Our rational modification via tannic acid etching and Cu^2^^+^ substitution overcomes conventional PB's core limitations, amplifying multi‐enzyme‐mimic activity, introducing pro‐angiogenic function, and enabling high‐capacity MaR1 loading [26, 27]. The central innovation of this work is the phase‐adaptive design, which spatiotemporally synchronizes acute nanozyme‐driven oxidative‐inflammatory control with delayed MaR1‐mediated mucosal regeneration, comprehensively addressing I/R's biphasic pathology—a key gap in traditional single‐target therapeutics [9, 57].

Biosafety evaluations confirmed no hepatic/renal toxicity or long‐term metal accumulation, supporting further translational development. Nevertheless, limitations remain: the study used murine models with pretreatment administration, which does not fully recapitulate clinical post‐injury intervention; XAS characterization is needed to fully resolve metal coordination environments; and single‐cell analyses are required to further delineate cell‐cell crosstalk in the injury microenvironment.

Ultimately, the MPB@TA‐Cu‐Ma nanoplatform addresses the unmet clinical need for comprehensive intestinal I/R therapy via dual‐phase, multi‐modal modulation. This work provides a promising translational candidate for I/R management, and a generalizable design strategy for nanotherapeutics targeting other ischemia‐reperfusion pathologies.

## 4 Conclusion

In summary, the phase‐adaptive nanoplatform MPB@TA‐Cu‐Ma demonstrated dual‐phase therapeutic efficacy against intestinal ischemia/reperfusion injury by sequentially coordinating antioxidative, anti‐inflammatory, and pro‐regenerative processes. Through integrated enzymatic catalysis, macrophage reprogramming, and angiogenic activation, it effectively bridged the temporal therapeutic gap between the injury and recovery phases. These findings highlight a promising strategy for precise and dynamic intervention in complex inflammatory disorders and provide a foundation for future translational development of phase‐adaptive nanotherapeutics.

## 5 Experimental Section

### 5.1 Synthesis of MPB@TA‐Cu‐Ma

The Prussian blue nanozyme (PB) product was synthesized as follows: Solution 1 was prepared by dissolving 1.2 g of citric acid and 135.15 mg of FeCl_3_·6H_2_O in 500 mL of double‐distilled water. Solution 2 was prepared by dissolving 1.2 g of citric acid and 211.2 mg of K_4_[Fe(CN)_6_] ·3H_2_O in 500 mL of double‐distilled water. Solution 2 was subjected to continuous magnetic stirring at 60°C, while Solution 1 was gradually added dropwise. The mixture turned a homogeneous blue, and stirring was maintained until the system reached room temperature. The PB was obtained by centrifugation (500,00 *×g*, 60 min) and stored at 4°C for future use. To introduce mesoporosity, the as‐synthesized PB was chemically etched with a 10 mg/mL tannic acid (TA) aqueous solution at 80°C for 2 h under vigorous agitation. The resulting porous framework (denoted as MPB@TA) was subsequently washed with deionized water and lyophilized. For copper ion substitution, MPB@TA was uniformly dispersed in a 10 mm copper (II) sulfate pentahydrate (CuSO_4_·5H_2_O) aqueous solution and magnetically stirred at 25°C for 24 h. The copper‐substituted product (MPB@TA‐Cu) was collected via centrifugation, rinsed thoroughly with ethanol, and vacuum‐dried. Finally, for Maresin 1(MaR1) (Caymanchem, USA) loading, the MPB@TA‐Cu nanoplatform was dispersed in pre‐chilled ethanol via bath ultrasonication for 10 min. Subsequently, a 1 mg/mL MaR1 ethanolic solution was added. The mixture was then incubated overnight at 4°C under shaking (120 rpm).

### 5.2 Scanning Electron Microscope (SEM)

Samples were sputter‐coated with platinum using an Edwards S150A system (Edwards, Burgess Hill, UK) to enhance conductivity before imaging. Subsequently, the coated samples were examined using a Hitachi SU8010 SEM (Hitachi, Tokyo, Japan) to capture high‐resolution images for detailed morphological analysis.

### 5.3 Nitrogen Adsorption–Desorption and Porosity Analysis

Nitrogen (N_2_) adsorption–desorption isotherms were recorded at 77.3 K using a Micromeritics ASAP 2460 analyzer. Before the measurements, the samples were degassed under vacuum at 303 K for 10 min. The specific surface areas were calculated via the multipoint Brunauer–Emmett–Teller (BET) method. Total pore volumes were determined from the single‐point adsorption quantity at a relative pressure (*P*/*P*_0_) approaching 1.0. The pore size distributions and differential pore volumes (d*V*/d*D*) were derived from the adsorption branches using the Barrett‐Joyner‐Halenda (BJH) model.

### 5.4 In Vitro Morphological Stability and Degradation Assay

To evaluate the structural stability under simulated intraperitoneal conditions, MPB@TA‐Cu (5 mg) was dispersed in phosphate‐buffered saline and incubated at 37°C under continuous shaking (150 rpm) in the dark. At predetermined intervals (0, 48, and 96 h), aliquots were collected and centrifuged (10000 rpm, 10 min). The precipitates were washed with deionized water three times to eliminate residual salts, redispersed, drop‐casted onto silicon wafers, and dried. After platinum sputter‐coating, the morphological evolution was observed using the aforementioned SEM.

### 5.5 X‐Ray Photoelectron Spectroscopy (XPS)

Samples were mounted onto a sample holder and introduced into the XPS chamber. The X‐ray source was activated to irradiate the sample, and photoelectron signals were collected and recorded. Both full‐spectrum and narrow‐spectrum scans were performed to obtain information on surface chemical composition and elemental valence states. Data were analyzed using specialized XPS software.

### 5.6 X‐Ray Diffraction (XRD)

The crystal structure of the samples was investigated using a D/MAX‐IIIC XRD instrument (Rigaku). Additionally, microstructural features were observed using a high‐resolution transmission electron microscope (Talos F200S, FEI) equipped with energy‐dispersive X‐ray spectroscopy (EDS).

### 5.7 Electron Spin Resonance (ESR)

ESR spectra were acquired using a Bruker EMX Electron Spin Resonance spectrometer. For superoxide anion detection, the xanthine‐xanthine oxidase (XO/XOD) reaction system was employed to generate these species, and 5,5‐dimethyl‐1‐pyrroline N‐oxide (DMPO) was used as a spin trap to form stable spin adducts. Similarly, hydroxyl radicals were generated using the TiO_2_/UV system, with DMPO serving as the spin trap. Nanozyme‐treated samples were subjected to ESR analysis to acquire and interpret the resulting spectra.

### 5.8 Inductively Coupled Plasma (ICP)

Nanomaterials (4 mg) were added to 2 mL of PBS preheated to 37°C and subjected to light‐protected agitation (100 rpm) in a constant‐temperature shaker. At predetermined time points (2, 12, 24, 72, 120, and 168 h), 1 mL aliquots of nanozyme suspensions were transferred to centrifuge tubes and labeled. After centrifugation to separate the supernatant from the precipitate, the supernatant was carefully aspirated, diluted, and acidified with nitric acid. The ICP instrument was calibrated before analysis, and the concentrations of Cu and Fe were recorded.

### 5.9 Tissue ICP‐MS Analysis

Fresh liver and kidney samples (*n* = 3/group) were rinsed in ice‐cold PBS, blotted dry, and accurately weighed. Tissues (∼100 mg) were microwave‐digested in Teflon vessels with 3 mL 70% HNO_3_ + 1 mL 30% H_2_O_2_ (program: 15 min ramp ‐ 180°C hold 30 min). Digests were diluted to 15 mL with deionized water, filtered (0.22 µm), and stored at 4°C for ICP‐MS analysis. Total Cu content was quantified by ICP‐MS (^6^ ^3^Cu isotope) with internal standard correction (^10^ ^3^Rh). Data reported as µg Cu/g wet weight.

### 5.10 Superoxide Dismutase (SOD)/ Catalase (CAT) Like Activity

The enzymatic activities of the SOD‐mimic (S0101S, Beyotime, China) and CAT‐mimic (BC0205, Solarbio, China) were evaluated. For the SOD‐mimic assay, superoxide anions were generated via the oxidation of xanthine, which exhibits a characteristic absorbance at 450 nm. The reaction mixture containing xanthine and xanthine oxidase was incubated in PBS at 37°C for 25 min, followed by the addition of nanozymes. The absorbance at 450 nm was then recorded to determine the SOD‐like activity. For the CAT‐mimic assay, nanomaterials were introduced into a hydrogen peroxide solution pre‐adjusted to the desired pH and ionic strength with PBS. The decomposition of hydrogen peroxide was monitored by UV spectrophotometry, and the CAT‐like activity was quantified according to the rate of absorbance change at the corresponding wavelength.

### 5.11 Density Functional Theory (DFT) Simulations

Spin‐polarized DFT calculations with GGA‐PBE were performed using VASP. Geometric optimization used uniform G‐centered k‐point meshes and Methfessel‐Paxton smearing, with convergence criteria of 1 meV/atom in energy, <10 meV/Å in force, and <0.03 GPa in stress. A 15 Å vacuum distance avoided periodic interactions, and DFT‐D3 corrections were applied. DMol3 with GGA‐PBE was used for structure optimization and charge distribution of 2TA‐Cu and 2TA‐Fe, with convergence thresholds of 10^−^^5^ Ha in energy, 0.001 Ha/Å in force, and 0.005 Å in displacement. ECPs with a 4.4 Å cutoff were used.

### 5.12 Liquid Chromatography‐Tandem Mass Spectrometry (LC‐MS/MS) Method

Chromatographic separation was performed on a Zorbax Eclipse Plus C18 column (2.1 × 150 mm, 1.8 µm, 9.5 nm; Agilent, Germany) at a flow rate of 0.3 mL min^−^^1^. Solvent A consisted of 0.1% acetic acid containing 5% solvent B, while solvent B was ACN/MeOH/acetic acid (800:150:1, v/v/v). The linear elution gradient was programmed as follows: 0–1.0 min, 21% B; 1.5 min, 26% B; 10 min, 51% B; 19 min, 66% B; 25.1–27.6 min, 98% B; returning to 21% B at 27.7 min and holding until 31.5 min. Mass spectrometry was conducted on a 6500 QTRAP (AB Sciex, Germany) in negative electrospray ionization (ESI^−^) mode. Nitrogen and zero air were utilized as the curtain/CAD and nebulizer/drying gases, respectively. Optimized source parameters included: ion spray voltage, −4500 V; source temperature, 475°C; curtain gas, 35 psi; GS1 and GS2, 60 psi each; and CAD gas, 15 psi. The probe was positioned at y = 0.250 cm and x = 0.550 cm with a 1.0–1.5 mm electrode protrusion. Detection was executed in scheduled selected reaction monitoring (SRM) mode (90 s detection window, 0.4 s cycle time) with individually optimized electronic parameters strictly for the quantification of MaR1 (monitoring precursor‐to‐product ion transitions at m/z 359.2 → 250.2 and 359.2 → 141.1).

### 5.13 In Vitro MaR1 Adsorption and Release Kinetics in Complete Culture Medium

A 10% (v/v) ethanol suspension was prepared by mixing 300 µL of MPB@TA‐Cu (2000 ppm), 150 µL of deionized water, and 50 µL of anhydrous ethanol in light‐resistant, low‐binding centrifuge tubes (Bioland, TP05‐015B). After adding 5 µg (50 uL) of MaR1 (MedChemExpress, HY‐116429), the tubes were nitrogen‐purged and incubated for 12 h at 4°C with shaking (120 rpm). Following centrifugation (12 000 rpm, 10 min, 4°C), the pellet was resuspended in 500 µL of complete culture medium containing 1 mm EDTA (Beyotime, ST1303‐50 g) and 50 µm BHT (Sangon Biotech, A501376). The sealed tubes were nitrogen‐purged and incubated in the dark at 37°C with shaking (120 rpm). At predetermined intervals (2, 6, 12, 24, 48, 72, and 96 h), samples were centrifuged, and 400 µL of supernatant was collected using low‐binding pipette tips, followed by immediate replenishment with 400 µL of fresh medium and nitrogen purging. Each collected supernatant was mixed with 800 µL of mass spectrometry‐grade methanol, vortexed, and centrifuged. Finally, 400 µL of the resulting supernatant was nitrogen‐purged and snap‐frozen in liquid nitrogen for LC‐MS analysis. The acquired LC‐MS readouts were multiplied by a dilution factor of 3 to calculate the cumulative MaR1 release over time.

### 5.14 Data Analysis for Drug Release

The cumulative release percentage of Mar1 was calculated by accounting for the drug quantities removed during all prior sampling intervals. The cumulative mass of the released Mar1 at time *t*(*M_t_*) was determined using the following equation:

$$M_{t}=C_{t}\times V_{t}+V_{s}\times \sum_{i=1}^{t-1}C_{i}$$

where *C_t_* is the actual concentration of Mar1 in the release medium at time *t*(after applying the dilution factor of 3 to the LC‐MS readouts), *V_t_* is the total volume of the release medium (0.5 mL), *V_s_* is the sampling volume removed at each time point (0.4 mL), and *C_i_* is the concentration of Mar1 at the previous time points. Subsequently, the cumulative release percentage (%) was calculated as follows: $\mathrm{Cumulative}\;\mathrm{Release}\;(\%)=(\frac{M_{t}}{M_{load}})\times 100$ where *M_load_* represents the initial actual loaded mass of Mar1 within the nanoparticles, obtained by subtracting the unadsorbed Mar1 in the 0 h supernatant from the total initial drug input (5 µg).

### 5.15 Preparation and Loading Efficiency of MaR1‐Loaded Nanozymes

MPB@TA‐Cu nanoparticles (0.3 mg/mL) and MaR1 (15 µg) were co‐incubated in 300 µL of pre‐chilled ethanol overnight at 4°C in the dark with shaking (120 rpm). The MaR1‐loaded nanozymes were isolated by centrifugation (10 000 rpm, 10 min). To evaluate loading efficiency, the supernatant containing unbound MaR1 was collected, appropriately diluted to fit the dynamic detection range, and quantified via a MaR1 ELISA kit (Item No. 10878, Cayman Chemical, USA) according to the manufacturer's instructions. The final unbound MaR1 concentration was calculated using the assay readout multiplied by the dilution factor. The loading capacity of different nanozymes for MaR1 was evaluated by comparing drug concentrations in the solution before and after centrifugation. The drug loading efficiency (%) of Maresin1 was calculated using:

$$R_{PB/TA/Cu}=\left(1-\frac{C_{PB/TA/Cu}}{C_{Mar1}}\right)\times 100\%$$

Where, *R_PB_* ​, *R_TA_* ​, and *R_Cu_​* denote the Mar1 adsorption rates of the PB, MPB@TA, and MPB@TA‐Cu groups. *C_PB_* ​, *C_TA_*, *C_Cu_​*​, and *C_Mar1_* denote the concentration of Maresin1 (pg/ml) in the supernatant of the PB, MPB@TA, MPB@TA‐Cu, and Mar1 groups, respectively.

The percentage increase in Mar1 adsorption efficiency for the MPB@TA and MPB@TA‐Cu groups compared to the PB group was calculated using:

$$P_{TA/Cu}=\frac{R_{TA/Cu}-R_{PB}}{R_{PB}}\times 100\%$$

Where, *P_TA_*: growth rate in Mar1 adsorption rate of the MPB@TA group compared to the PB group. *P_Cu_*: growth rate in Mar1 adsorption rate of the MPB@TA‐Cu group compared to the PB group.

### 5.16 Dialysis‐Based MaR1 Release Profile in PBS Buffer

MaR1 release from MPB@TA‐Cu was evaluated using 3.5 kDa molecular weight cut‐off (MWCO) dialysis tubing pre‐equilibrated in PBS containing 0.01% BHT, 1 mM EDTA, 0.5% BSA, and 0.01% Tween‐20. The tubing was loaded with 1 mL of a 2 mg/mL MPB@TA‐Cu‐Ma nanosuspension (2000 ppm; equivalent MaR1 concentration: 750 ng/mL) and immersed in 20 mL of pre‐warmed (37°C) release medium under light‐protected agitation (100 rpm). At predetermined time points (0.5, 1, 2, 4, 8, 12, 24, 48, and 72 h), the entire release medium was collected and replaced with fresh pre‐warmed medium. Samples were centrifuged at 12000 × g for 5 min, diluted 10‐fold, and the released MaR1 concentration was quantified using a Maresin‐1 ELISA kit (501150, Cayman Chemical, USA).

### 5.17 Cell Culture and Hypoxia/Reoxygenation(H/R) Cell Modeling

RAW264.7 cells (murine macrophage cell line, RRID: CVCL_0493; FH0328, FuHeng Biology, Shanghai, China), MODE‐K cells (murine intestinal epithelial cell line, RRID: RRID: CVCL_B4FG; HTX2023, Otwo Biotech Inc., Shenzhen, China), and HUVECs (immortalized human umbilical vein endothelial cells, RRID: CVCL_E5ZU; TCH‐C406‐UD, Haixing Biosciences, China) were obtained in 2025. All cell lines were routinely tested for mycoplasma contamination and were found to be negative. These cells were cultured in their respective complete media: RAW264.7 cells in RPMI 1640, MODE‐K cells in DMEM, and HUVECs in ScienCell ECM endothelial cell culture medium, all supplemented with 10% serum. Cultures were maintained at 37°C in a 5% CO_2_ incubator. For the H/R treatment, which consists of exposing cells to a cycle of hypoxia followed by reoxygenation, the media of RAW264.7 and MODE‐K cells were replaced with glucose‐free DMEM 12 h prior to the treatment. Subsequently, the cells were incubated in a hypoxic environment at 37°C for 6 h. After the hypoxic period, the media were changed to DMEM containing 10% serum, and the cells underwent reoxygenation under routine culture conditions at 37°C for 6 h.

### 5.18 Cell Proliferation Activity Assay

Cell Counting Kit‐8 (CCK‐8, APExBIO, USA) was used to assess the effects of nanozymes (PB, MPB@TA, MPB@TA‐Cu) on RAW 264.7 and MODE‐K cell viability. First, cells pre‐cultured in 96‐well plates (37°C, 5% CO_2_, 24 h) were treated with 10 µL of nanozymes at gradient concentrations (0, 50, 100, 200, 300, 400 ppm) for 24 h. After adding CCK‐8 (10 µL/well, 37°C, 1 h), absorbance at 450 nm (Bio‐Tek) was measured. Next, cells were exposed to 10 µL of 200 ppm nanozymes for 24, 48, and 72 h, with CCK‐8 detection as above to evaluate time‐dependent effects.

### 5.19 Transmission Electron Microscopy (TEM) Analysis

For ultrastructural analysis of macrophages:Macrophage samples were fixed with 2.5% glutaraldehyde (AR grade, Sinopharm Chemical Reagent Co., Ltd.) in PBS (0.1 m, pH 7.2–7.4, Fortune Chemical Reagent Co., Ltd., Tianjin), post‐fixed in 1% osmium tetroxide (Zhongjing Technology Co., Ltd., Beijing), and dehydrated via an acetone gradient (AR grade, 30% to 100%, Xilong Scientific Co., Ltd.) with triple 100% acetone exchanges. After infiltration with acetone/Epon‐812 resin mixtures (3:1 to 1:3) and embedding in pure Epon‐812 resin, ultrathin sections (60–90 nm) were cut, mounted on copper grids, and stained with uranyl acetate (Zhongjing Technology Co., Ltd., Beijing; 10–15 min) and lead citrate (Zhongjing Technology Co., Ltd., Beijing; 1–2 min). Imaging was performed using a JEM‐1400FLASH TEM (JEOL Ltd., Japan), with low‐magnification grid screening followed by high‐resolution acquisition of pathological features. For characterization of Prussian blue nanozymes (PB nanozymes): PB nanozyme samples were dispersed in analytical‐grade absolute ethanol at 0.1 mg/mL, followed by ice‐bath ultrasonic treatment (300 W, 5 min) to prevent aggregation. A 5 µL aliquot was dropped onto a 300‐mesh carbon‐supported copper grid (Ted Pella, USA) and air‐dried in a desiccator at room temperature for 2 h. TEM imaging was performed using a JEM‐2100 TEM (JEOL, Japan) at 200 kV, with images acquired at 5000×–1 00 000× magnifications to analyze morphology (shape, size distribution) and structural features (e.g., lattice fringes). Size distribution was analyzed via ImageJ software using particles from random images.

### 5.20 ELISA Assay

Cell culture supernatants and mouse serum samples were collected under the indicated conditions. The levels of IL‐1β, IL‐6, TNF‐α, and IL‐18 were quantified using Mouse IL‐1 beta ELISA Kit (Proteintech, KE10003, China), Mouse TNF‐alpha ELISA Kit (Proteintech, KE10002, China), Mouse IL‐6 ELISA Kit (Proteintech, KE10007, China), and Mouse IL‐18 ELISA Kit (Beyotime Biotechnology, PI553, China) according to the manufacturers’ instructions. Absorbance was measured at 450 nm using a microplate reader.

### 5.21 Apoptosis Detection

Cells were treated as described for modeling and drug intervention. Apoptosis was detected using the Annexin V‐FITC/PI Apoptosis Detection Kit (Beyotime Biotechnology, C1062M, China) according to the manufacturer's instructions. After washing with cold PBS, MODE‐K cells were resuspended in binding buffer and incubated with Annexin V‐FITC and PI solution at room temperature for 15 min in the dark. Apoptosis was analyzed by flow cytometry using a CytoFLEX flow cytometer (Beckman Coulter, USA).

### 5.22 Scratch Wound Healing Assay

MODE‐K murine intestinal epithelial cells were cultured in DMEM with 10% FBS and 1% penicillin/streptomycin (37°C, 5% CO_2_). After hypoxia/reoxygenation (H/R) treatment, a standardized scratch was created in confluent monolayers using a 200 µL sterile pipette tip. Cells were washed with PBS and incubated in 1% FBS medium. Wound closure was quantified at 0, 24, and 48 h via phase‐contrast microscopy (Zeiss, Germany). The wound area was quantified using ImageJ software by threshold‐based segmentation.

### 5.23 In Vitro Angiogenesis Test

HUVEC were preincubated with 100 µL/mL of serum‐free medium containing MPB@TA‐Cu, MaR1, or MPB@TA‐Cu‐Ma for 12 h. Matrigel (Corning, USA) was added to the 24‐well plate, which was then incubated at 37°C for 30 min until the Matrigel solidified. HUVECs (3 × 10^4^ cells/well) were seeded onto the solidified Matrigel matrix and cultured in ECM. After 6 h of incubation (5% CO_2_, 37°C), the cells were imaged using a microscope (Zeiss, Oberkochen, Germany).

### 5.24 ROS Detection

Cells or frozen tissue sections were incubated in DCFH‐DA (Beyotime Biotechnology, S0033S, China) solution at 37°C for 30 min. Subsequently, they were washed three times with serum‐free cell culture medium. ROS levels were then detected by immunofluorescence or flow cytometry using a CytoFLEX flow cytometer (Beckman Coulter, USA).

### 5.25 Immunohistochemistry Studies

The intestinal tissue was fixed with 4% paraformaldehyde, embedded in paraffin, and sectioned. After dewaxing and rehydration, antigen retrieval was performed using citrate buffer (pH 6.0)/ Tris‐EDTA (pH 9.0) under high‐pressure heat treatment (95°C, 20 min), followed by blocking nonspecific binding sites with 5% BSA. The sections were then incubated with primary antibodies against TNF‐α (Proteintech, 26405‐1‐AP, 1:200, RRID:AB_2918102), GSDMD‐N (Immunoway, YT7991, 1:100, RRID:AB_3663000), Caspase‐1 (Biodragon, BD‐PT5743, 1:200), NLPR3 (MCE, HY‐P80246, 1:200), GSTM1 (Proteintech, 12412‐1‐AP, 1:200, RRID:AB_2115925), NOX1 (Biodragon, BD‐PT2820, 1:200), VEGF (Proteintech, 19003‐1‐AP, 1:200, RRID:AB_2212657), ACE (Proteintech, 24743‐1‐AP, 1:200, RRID:AB_2879701), DSG1 ( Biodragon, BD‐PT5886,1:200) and DEFA1 (Biodragon, BD‐PT1322, 1:200) overnight at 4°C, and followed by incubation with biotin‐conjugated anti‐rabbit antibody or anti‐goat antibody. Finally, the OD values of positive areas within the small intestine tissues were measured using ImageJ software.

### 5.26 Immunofluorescence Detection

Intestinal tissue was fixed with 4% paraformaldehyde, embedded in paraffin, and sectioned. Blocking treatments were performed, and the sections were incubated overnight at 4°C with primary antibodies against CD86 (Proteintech, 13395‐1‐AP, 1:200, RRID:AB_2074882), CD206 (Cell Signaling Technology, 24595S, 1:100), α‐SMA (Proteintech, 14395‐1‐AP, 1:200, RRID:AB_2223009), CD31 (Cell Signaling Technology, 15585T, 1:200), and Occludin (Abcam, ab216327, 1:200, RRID:AB_2737295). The next day, after washing with PBS, the sections were incubated with Alexa Fluor 594 or Alexa Fluor 488‐labeled secondary antibodies at room temperature in the dark for 30 min. DAPI (Beyotime Biotechnology, C1099, China) counterstaining was used to visualize the nuclei. Immunofluorescent images were acquired via an Olympus microscope (IXplore SpinSR, Olympus Corporation, Tokyo, Japan) and analyzed using ImageJ software.

### 5.27 Western Blotting Analysis

Cells were lysed in RIPA buffer containing protease inhibitors, and cell culture supernatants were collected and concentrated via centrifugation at 12 000 rpm for 15 min. Proteins were separated by 10%‐12% SDS‐PAGE gels and then transferred to PVDF membranes. The membranes were blocked with 5% BSA in TBST for 2 h at room temperature and then incubated overnight at 4°C with primary antibodies against NLRP3 (Proteintech, 30109‐1‐AP, 1:1000, RRID:AB_3086231),Casp1 (Proteintech, 81482‐1‐RR, 1:1000, RRID:AB_2935555),Cleaved‐casp1 (Cell Signaling Technology, 89332T, 1:1000), GSDMD (Abcam, ab209845, 1:1000, RRID:AB_2783550), cleaved‐GSDMD (Cell Signaling Technology, 10137s, 1:1000), and β‐actin (Sigma‐Aldrich, A5441, 1:1000, RRID:AB_476744). This was followed by incubation with HRP‐conjugated secondary antibodies (Abcam, ab6721, RRID: AB_955447) for 2 h at room temperature. The immunoreactive bands were visualized using enhanced chemiluminescence (Millipore, Burlington, MA), and images were obtained and analyzed using a fluorescent imaging system (ProteinSimple, Santa Clara, CA, USA) and ImageJ software.

### 5.28 Animal Experiments

The mice were housed in a pathogen‐free animal facility. All animal experiments were subjected to approval by the Animal Ethics Committee of the Second Hospital of Chongqing Medical University (IACUC‐SAHCQMU‐2024‐00101). C57BL/6 (RRID: IMSR_JAX:000664) *Gsdmd* ^fl/fl^ (RRID: IMSR_NM‐CKO‐190060) and *Cx3cr1* ^Cre/+^ (RRID: IMSR_EM:06349) mice were procured from Gempharmatech Co. Ltd. (Nanjing, China). *Gsdmd* ^fl/fl^ mice were crossed with *Cx3cr1* ^Cre/+^ mice to obtain macrophage‐specific *Gsdmd*‐knockout mice (*Cx3cr1* ^Cre/+^; *Gsdmd* ^fl/fl^; abbreviated as *Gsdmd* ^ΔMΦ^).

### 5.29 Dose‐Escalation Study in Intestinal I/R Injury Model

To determine the optimal therapeutic dose of MPB@TA‐Cu‐Ma, a dose‐escalation study was conducted in a murine intestinal ischemia/reperfusion (I/R) injury model. Mice were intraperitoneally administered MPB@TA‐Cu‐Ma at concentrations of 0.2, 2, or 4 mg/mL (100 µL per mouse, corresponding to 15, 150, and 300 ng MaR1, respectively). At 6 h post‐reperfusion, intestinal tissues were collected to evaluate the acute inflammatory response and apoptosis. TNF‐α (Proteintech, 26405‐1‐AP, 1:200, RRID: AB_2918102) expression was assessed by immunohistochemistry, and apoptotic cells were detected using TUNEL staining (Beyotime Biotechnology, C1099, China). At 96 h post‐reperfusion, tissues were harvested to assess epithelial barrier recovery by examining the expression of the tight junction protein Occludin (Abcam, ab216327, 1:200, RRID: AB_2737295) using immunofluorescence staining.

### 5.30 Isolation and Sorting of Macrophages From Mouse Spleens Following I/R Surgery

Spleens from I/R‐treated mice were immediately placed in pre‐cooled PBS, and single‐cell suspensions were prepared by mechanical grinding through a 40 µm cell strainer; red blood cells were removed with lysis buffer (155 mm NH_4_Cl, 10 mm KHCO_3_, 0.1 mm EDTA) for 5 min, followed by centrifugation at 300 g for 5 min at 4°C, with pellets resuspended in 2% FBS‐PBS. For cell sorting, lineage depletion was first performed by incubating the cell suspension with a biotin‐conjugated antibody cocktail (Anti‐Ly6G‐biotin, Thermo, 13‐5931‐85, 1:100, RRID: AB_466801; Anti‐CD3ε‐biotin, Thermo, 13‐0031‐85, 1:200, RRID:AB_466320; Anti‐CD19‐biotin, Thermo, 13‐0193‐85, 1:200, RRID:AB_657658; Anti‐NK1.1‐biotin, Thermo, 13‐5941‐85, 1:200, RRID:AB_466805) on ice for 15 min, then negative selection was conducted by adding anti‐biotin microbeads (Miltenyi, 130‐090‐485, RRID:AB_244365), rotating at 4°C for 10 min, and passing through an MS column to collect the flow‐through; subsequently, for macrophage positive sorting, anti‐CD11b microbeads (Miltenyi, 130‐049‐601, RRID:AB_2927377) were added to the flow‐through, incubated at 4°C for 15 min, and passed through an MS column again. The eluted CD11b^+^ macrophages were collected first, then immediately lysed with Trizol to extract RNA, and the extracted RNA was used for qPCR validation.

### 5.31 Quantitative Real‐time Polymerase Chain Teaction (qPCR) Analysis

Total RNA was isolated from the isolated and sorted splenic macrophages using Trizol reagent (R1100, Solarbio). After measuring RNA concentration and purity, cDNA was synthesized via reverse transcription. Fluorescent quantitative PCR was performed according to the qPCR kit (AG11728, Accurate Biology) manufacturer's instructions to determine the mRNA levels of GSDMD, with GAPDH serving as the internal reference gene. Data were analyzed using the 2^‐ΔΔCt^ method. The qPCR primers were synthesized by Sangon Biotech Co., Ltd (Shanghai), with the following sequences: GSDMD forward primer: 5′‐CCATCGGCCTTTGAGAAAGTG‐3′; GSDMD reverse primer: 5′‐ACACATGAATAACGGGGTTTCC‐3′; GAPDH forward primer: 5′‐GAAGGTGGTGAAGCAGGCATC‐3′; and GAPDH reverse primer: 5′‐GTGGGAGTTGCTGTTGAAGTC‐3′.

### 5.32 Mouse Small Intestine I/R Injury Model

Adult male C57BL/6J mice were obtained from Hunan SJA Laboratory Animal Co. Ltd. and bred at The Second Hospital of Chongqing Medical University. Mice were housed under standard conditions, and all experimental protocols were approved. Based on previous studies, the intestinal ischemia/reperfusion (I/R) animal model was constructed [66]. Mice were fasted for 12 h before surgery, injected with amobarbital sodium, and the superior mesenteric artery (SMA) was occluded to induce intestinal ischemia. After 45 min, the clamp was removed to restore blood perfusion. Mice were euthanized 6 h post‐reperfusion. For the MaR1/MPB@TA‐Cu/MPB@TA‐Cu‐Ma conditioning group, required doses of agents were administered intraperitoneally 45 min before surgery. (IACUC‐SAHCQMU‐2024‐00101).

### 5.33 Tissues HE Staining

Small intestinal tissues were fixed in 4% paraformaldehyde for 24 h, embedded in paraffin, and stained with HE after sectioning. The histopathological score was determined via Chiu's scoring system [67].

### 5.34 FD‐4 Intestinal Mucosal Barrier Function Test

To evaluate intestinal mucosal barrier permeability after intestinal ischemia‐reperfusion (I/R) in mice, 4 kD FITC‐Dextran (FD‐4, Sigma‐Aldrich, USA) was used. Mice were gavaged with FD‐4 solution at the onset of ischemia. After 4 h of reperfusion, blood was collected and centrifuged to obtain the supernatant, which was analyzed for fluorescence intensity to calculate the FD‐4 content using a standard curve.

### 5.35 Serum Biochemical Analysis

Mouse blood was collected and centrifuged at 3000 rpm at 4°C for 10 min to separate serum (with precautions to avoid hemolysis, and the serum was analyzed immediately after preparation). Serum levels of ALT (Alanine Aminotransferase), AST (Aspartate Aminotransferase), BUN (Blood Urea Nitrogen), and CREA (Creatinine) were then determined using Solarbio kits (BC1555 for ALT, BC1565 for AST) and Beyotime kits (S0574S for BUN, S0291S for CREA) following the manufacturers’ instructions.

### 5.36 Transcriptome Sequencing and Analysis

RNA was extracted from mouse intestinal tissue samples, and poly‐A selection was performed to enrich eukaryotic mRNA using the NEBNext Poly(A) mRNA Magnetic Isolation Module. The purified mRNA was fragmented and reverse‐transcribed into cDNA, followed by library construction with the Illumina Stranded mRNA Prep Kit. The libraries were subjected to paired‐end sequencing (2 × 150 bp) on the Illumina NovaSeq 6000 platform (Illumina, San Diego, USA). For sequence quality control, fastp software (version 0.23.4) was used to trim adapters and filter low‐quality reads (Q20< 90%). Clean reads were aligned to the mouse reference genome (GRCm39) using STAR (version 2.7.10a) with default parameters. Gene expression quantification was performed using featureCounts (version 2.0.3) based on the GENCODE mouse annotation (M32). Differential expression analysis was conducted with DESeq2 (version 1.38.3), applying thresholds of |log2 fold change| > 1 and adjusted *p*‐value < 0.05. All data analyses were executed on the Majorbio Cloud Platform (https://cloud.majorbio.com) [68].

### 5.37 Statistical Analysis

Statistical analyses were performed using GraphPad Prism. Data are presented as mean ± SEM, and sample sizes (n) are indicated in the figure legends. Comparisons between two groups were performed using unpaired two‐tailed Student's *t*‐test or the non‐parametric Mann–Whitney U test, as appropriate. Multiple‐group comparisons were conducted using one‐way or two‐way ANOVA followed by Tukey's post hoc test, or the Kruskal–Wallis test followed by Dunn's multiple comparisons test for non‐parametric data. Ordinal data (e.g., Chiu scores) were analyzed using non‐parametric tests. *P* < 0.05 was considered statistically significant.

## Conflicts of Interest

The authors declare no conflicts of interest.

## Supporting information




**Supporting File**: advs75059‐sup‐0001‐SuppMat.docx.

## Data Availability

Data will be publicly available upon acceptance.

## References

[1] J. S. Boomer , K. To , K. C. Chang , et al., “Immunosuppression in Patients Who Die of Sepsis and Multiple Organ Failure,” Jama 306, no. 23 (2011): 2594–2605, 10.1001/jama.2011.1829. 22187279 PMC3361243

[2] M. Singer , C. S. Deutschman , C. W. Seymour , et al., “The Third International Consensus Definitions for Sepsis and Septic Shock (Sepsis‐3),” Jama 315, no. 8 (2016): 801–810, 10.1001/jama.2016.0287. 26903338 PMC4968574

[3] T. Zhang , D. Fan , K. Qin , et al., “Itaconate Facilitates Methane‐induced Nrf_2_ Pathway Activation for Mitigating Liver Ischemia and Reperfusion Injury,” Iliver 4, no. 1 (2025): 100144, 10.1016/j.iliver.2025.100144. 40636783 PMC12212682

[4] F. Deng , Z.‐B. Lin , Q.‐S. Sun , et al., “The Role of Intestinal Microbiota and Its Metabolites in Intestinal and Extraintestinal Organ Injury Induced by Intestinal Ischemia Reperfusion Injury,” International Journal of Biological Sciences 18, no. 10 (2022): 3981–3992, 10.7150/ijbs.71491. 35844797 PMC9274501

[5] W. Shi , X. Zhou , X. Li , et al., “Human Umbilical Cord Mesenchymal Stem Cells Protect Against Renal Ischemia‐Reperfusion Injury by Secreting Extracellular Vesicles Loaded With miR‐148b‐3p That Target Pyruvate Dehydrogenase Kinase 4 to Inhibit Endoplasmic Reticulum Stress at the Reperfusion Stages,” International Journal of Molecular Sciences 24, no. 10 (2023): 8899. 37240246 10.3390/ijms24108899 PMC10219505

[6] S. M. Davidson , P. Ferdinandy , I. Andreadou , et al., “Multitarget Strategies to Reduce Myocardial Ischemia/Reperfusion Injury,” Journal of the American College of Cardiology 73, no. 1 (2019): 89–99, 10.1016/j.jacc.2018.09.086. 30621955

[7] C. Barnig , G. Lutzweiler , M. Giannini , et al., “Resolution of Inflammation After Skeletal Muscle Ischemia‐Reperfusion Injury: A Focus on the Lipid Mediators Lipoxins, Resolvins, Protectins and Maresins,” Antioxidants 11, no. 6 (2022): 1213. 35740110 10.3390/antiox11061213 PMC9220296

[8] N. Chaudhary , M. Arif , S. Shafi , S. P. Kushwaha , and P. Soni , “Emerging Role of Natural Bioactive Compounds in Navigating the Future of Liver Disease,” Iliver 4, no. 1 (2025): 100140, 10.1016/j.iliver.2024.100140. 40636787 PMC12212691

[9] L. Zhang , Z. Qin , H. Sun , et al., “Nanoenzyme Engineered Neutrophil‐derived Exosomes Attenuate Joint Injury in Advanced Rheumatoid Arthritis via Regulating Inflammatory Environment,” Bioactive Materials 18 (2022): 1–14, 10.1016/j.bioactmat.2022.02.017. 35387158 PMC8961303

[10] W. Zhang , S. Hu , J.‐J. Yin , et al., “Prussian Blue Nanoparticles as Multienzyme Mimetics and Reactive Oxygen Species Scavengers,” Journal of the American Chemical Society 138, no. 18 (2016): 5860–5865, 10.1021/jacs.5b12070. 26918394

[11] J. Wen , Z. Zhao , F. Fang , et al., “Prussian Blue Nanoparticle‐Entrapped GelMA Gels Laden With Mesenchymal Stem Cells as Prospective Biomaterials for Pelvic Floor Tissue Repair,” International Journal of Molecular Sciences 24, no. 3 (2023): 2704, 10.3390/ijms24032704. 36769027 PMC9916949

[12] I. Nagaoka , H. Tamura , and J. Reich , “Therapeutic Potential of Cathelicidin Peptide LL‐37, an Antimicrobial Agent, in a Murine Sepsis Model,” International Journal of Molecular Sciences 21, no. 17 (2020): 5973. 32825174 10.3390/ijms21175973 PMC7503894

[13] P. Devant , E. Borsic , E. M. Ngwa , et al., “Gasdermin D Pore‐forming Activity Is Redox‐sensitive,” Cell Reports 42, no. 1 (2023): 112008, 10.1016/j.celrep.2023.112008. 36662620 PMC9947919

[14] Z. Qu , J. Zhou , Y. Zhou , et al., “Mycobacterial EST12 Activates a RACK1–NLRP3–Gasdermin D Pyroptosis–IL‐1β Immune Pathway,” Science Advances 6, no. 43 (2020), 10.1126/sciadv.aba4733. PMC7608829 33097533

[15] T. J. Barrett , “Macrophages in Atherosclerosis Regression,” Arteriosclerosis, Thrombosis, and Vascular Biology 40, no. 1 (2020): 20–33, 10.1161/ATVBAHA.119.312802. 31722535 PMC6946104

[16] U. Patel , S. Rajasingh , S. Samanta , T. Cao , B. Dawn , and J. Rajasingh , “Macrophage Polarization in Response to Epigenetic Modifiers During Infection and Inflammation,” Drug Discovery Today 22, no. 1 (2017): 186–193, 10.1016/j.drudis.2016.08.006. 27554801 PMC5226865

[17] F. Zhang , H. Wang , X. Wang , et al., “TGF‐β Induces M2‐Like Macrophage Polarization via SNAIL‐Mediated Suppression of a Pro‐Inflammatory Phenotype,” Oncotarget 7, no. 32 (2016): 52294–52306, 10.18632/oncotarget.10561. 27418133 PMC5239552

[18] L. Yan , J. Wang , X. Cai , et al., “Macrophage Plasticity: Signaling Pathways, Tissue Repair, and Regeneration,” MedComm 5, no. 8 (2020): 658, 10.1002/mco2.658. PMC11292402 39092292

[19] N. Saito‐Sasaki , Y. Sawada , and M. Nakamura , “Maresin‐1 and Inflammatory Disease,” International Journal of Molecular Sciences 23, no. 3 (2022): 1367, 10.3390/ijms23031367. 35163291 PMC8835953

[20] H. Zhang , J. Wang , J. Shen , et al., “Prophylactic Supplementation With Bifidobacterium infantis or Its Metabolite Inosine Attenuates Cardiac Ischemia/Reperfusion Injury,” Imeta 3, no. 4 (2024): 220, 10.1002/imt2.220. PMC11316933 39135700

[21] T. Li , H. Zeng , W. Xian , et al., “Maresin1 Alleviates Liver Ischemia/Reperfusion Injury by Reducing Liver Macrophage Pyroptosis,” Journal of Translational Medicine 21, no. 1 (2023): 472, 10.1186/s12967-023-04327-9. 37455316 PMC10351145

[22] D. S. Im , “Maresin‐1 Resolution With RORα and LGR6,” Progress in Lipid Research 78 (2020): 101034, 10.1016/j.plipres.2020.101034. 32360520

[23] Y.‐H. Han , K.‐O. Shin , J.‐Y. Kim , et al., “A Maresin 1/RORα/12‐Lipoxygenase Autoregulatory Circuit Prevents Inflammation and Progression of Nonalcoholic Steatohepatitis,” Journal of Clinical Investigation 129, no. 4 (2019): 1684–1698, 10.1172/JCI124219. 30855276 PMC6436872

[24] J. A. Castor‐Macias , J. A. Larouche , E. C. Wallace , et al., “Maresin 1 Repletion Improves Muscle Regeneration After Volumetric Muscle Loss,” Elife 12 (2023): 86437, 10.7554/eLife.86437. PMC10807862 38131691

[25] S.‐Y. Yin , G. Song , Y. Yang , et al., “Persistent Regulation of Tumor Microenvironment via Circulating Catalysis of MnFe_2_O_4_@ Metal–Organic Frameworks for Enhanced Photodynamic Therapy,” Advanced Functional Materials 29, no. 25 (2019): 1901417, 10.1002/adfm.201901417.

[26] W. Baer‐Dubowska , H. Szaefer , A. Majchrzak‐Celinska , and V. Krajka‐Kuzniak , “Tannic Acid: Specific Form of Tannins in Cancer Chemoprevention and Therapy‐Old and New Applications,” Current Pharmacology Reports 6 (2020): 28–37, 10.1007/s40495-020-00211-y.

[27] S. R. Abulateefeh and M. O. Taha , “Enhanced Drug Encapsulation and Extended Release Profiles of Calcium–Alginate Nanoparticles by Using Tannic Acid as a Bridging Cross‐Linking Agent,” Journal of Microencapsulation 32, no. 1 (2015): 96–105, 10.3109/02652048.2014.985343. 25413187

[28] T. Xue , F. Liu , B. Lu , et al., “A Prussian Blue Analog as a Decorporation Agent for the Simultaneous Removal of Cesium and Reactive Oxygen Species,” Nanoscale Advances 5, no. 20 (2023): 5661–5670, 10.1039/D3NA00388D. 37822904 PMC10563846

[29] B. Qi , Q. Xu , Y. Cao , and Z. Xiao , “Photothermal and Catalytic Performance of Multifunctional Cu‐Fe Bimetallic Prussian Blue Nanocubes With the Assistance of Near‐Infrared Radiation,” Nanomaterials 13, no. 13 (2023): 1897, 10.3390/nano13131897. 37446413 PMC10343283

[30] S. B. Nagarajan , A. Jayaraman , and S. Ramakrishnan , “Theranostic Scope of Monometallic Selenium and Titanium Dioxide Nanoparticles in Biomedicine: A Review,” Health Care Science 3, no. 4 (2024): 215–231, 10.1002/hcs2.109. 39220427 PMC11362656

[31] S. B. Nagarajan , S. Ramakrishnan , and A. Jayaraman , “Theranostic Aspects of Palladium‐Based Bimetallic Nanoparticles in Biomedical Field: A State‐of‐the‐Art,” Health Care Science 3, no. 3 (2024): 181–202, 10.1002/hcs2.96. 38947365 PMC11212303

[32] M. P. Beyer , L. A. Videla , C. Farías , and R. Valenzuela , “Potential Clinical Applications of Pro‐Resolving Lipids Mediators From Docosahexaenoic Acid,” Nutrients 15, no. 15 (2023): 3317. 37571256 10.3390/nu15153317 PMC10421104

[33] C. N. Serhan , N. Chiang , J. Dalli , and B. D. Levy , “Lipid Mediators in the Resolution of Inflammation,” Cold Spring Harbor Perspectives in Biology 7, no. 2 (2015): a016311, 10.1101/cshperspect.a016311. PMC4315926 25359497

[34] Z. Huang , W. Wang , L. Shu , et al., “Explicating the Publication Paradigm by Bibliometric Approaches: A Case of Interplay Between Nanoscience and Ferroptosis,” Health Care Science 1, no. 2 (2022): 93–110, 10.1002/hcs2.5. 38938888 PMC11080826

[35] C. Chen , H. Yang , X. Yang , and Q. Ma , “Tannic Acid: A Crosslinker Leading to Versatile Functional Polymeric Networks: A Review,” RSC Advances 12, no. 13 (2022): 7689–7711, 10.1039/D1RA07657D. 35424749 PMC8982347

[36] C. Marquez , F. G. Cirujano , S. Smolders , et al., “Metal Ion Exchange in Prussian Blue Analogues: Cu (II)‐exchanged Zn–Co PBAs as Highly Selective Catalysts for A3 Coupling,” Dalton Transactions 48, no. 12 (2019): 3946–3954, 10.1039/C9DT00388F. 30829365

[37] W. S. Cheon , J. Bu , S. Jung , et al., “Enhanced Oxygen Evolution Reaction of 2‐Dimensional Metal‐Organic Frameworks With Tunable Nitrogen Functionalities by Ion Beam Sputtering,” Chemical Engineering Journal 489 (2024): 151004, 10.1016/j.cej.2024.151004.

[38] K. Fan , H. Wang , J. Xi , et al., “Optimization of Fe_3_O_4_ Nanozyme Activity via Single Amino Acid Modification Mimicking an Enzyme Active Site,” Chemical Communications 53 (2017): 424–427, 10.1039/C6CC08542C. 27959363

[39] Y. Wang , X. Wang , and M. Antonietti , “Polymeric Graphitic Carbon Nitride as a Heterogeneous Organocatalyst: From Photochemistry to Multipurpose Catalysis to Sustainable Chemistry,” Angewandte Chemie International Edition 51, no. 1 (2012): 68–89, 10.1002/anie.201101182. 22109976

[40] S. Liu , J. Tian , L. Wang , et al., “Hydrothermal Treatment of Grass: A Low‐Cost, Green Route to Nitrogen‐Doped, Carbon‐Rich, Photoluminescent Polymer Nanodots as an Effective Fluorescent Sensing Platform for Label‐Free Detection of Cu(II) Ions,” Advanced Materials 24, no. 15 (2012): 2037–2041, 10.1002/adma.201200164. 22419383

[41] L. Zhang , M. Fang , C. Yang , F. Zhang , Z. Lu , and H. Cao , “In‐situ Coordination Assembly of Polyphenol With Cage‐Like Prussian Blue as an Ecofriendly Nanocarrier for Site‐specific Pesticide Delivery and Sustained Pest Control,” Journal of Cleaner Production 434 (2024): 139732, 10.1016/j.jclepro.2023.139732.

[42] A. Zeb , S. Sahar , S.‐Y. Lv , et al., “Engineering at Subatomic Scale: Achieving Selective Catalytic Pathways via Tuning of the Oxidation States in Functionalized Single‐Atom Quantum Catalysts,” Small 18, no. 34 (2022): 2202522, 10.1002/smll.202202522. 35896869

[43] G. Zhang , H. Chen , G. Yang , and H. Fu , “Preparation of in Situ ZIF‐9 Grown on Sodium Alginate/Polyvinyl Alcohol Hydrogels for Enhancing Cu (II) Adsorption From Aqueous Solutions,” Journal of Inorganic and Organometallic Polymers and Materials 32, no. 12 (2022): 4576–4588, 10.1007/s10904-022-02463-1.

[44] M. Z. Hussain , B. van der Linden , Z. Yang , et al., “Bimetal–Organic Framework Derived Multi‐Heterostructured TiO_2_/Cu X O/C Nanocomposites With Superior Photocatalytic H_2_ Generation Performance,” Journal of Materials Chemistry A 9, no. 7 (2021): 4103–4116, 10.1039/D0TA10853G.

[45] A. A. Mashentseva , N. Seitzhapar , M. Barsbay , et al., “Adsorption Isotherms and Kinetics for Pb (II) Ion Removal From Aqueous Solutions With Biogenic Metal Oxide Nanoparticles,” RSC Advances 13, no. 38 (2023): 26839–26850, 10.1039/D3RA05347D. 37692348 PMC10483273

[46] Y. Zeng , Z. Li , C. Gao , et al., “Electrical Conductivity and Temperature Sensitivity of Cu/Mo Co‐Modified YFeoO Ceramics,” Processing and Application of Ceramics 15, no. 2 (2021): 195–201, 10.2298/PAC2102195Z.

[47] H. Niu , S. Liu , Y. Cai , F. Wu , and X. Zhao , “MOF Derived Porous Carbon Supported Cu/Cu_2_O Composite as High Performance Non‐Noble Catalyst,” Microporous and Mesoporous Materials 219 (2016): 48–53, 10.1016/j.micromeso.2015.07.027.

[48] M. Song , J. Han , Y. Wang , L. Chen , Y. Chen , and X. Liao , “Effects and Mechanisms of Cu Species in Fe‐MOFs on Fenton‐Like Catalytic Activity and Stability,” ACS Applied Materials & Interfaces 15, no. 30 (2023): 36201–36213, 10.1021/acsami.3c05928. 37464747

[49] B. Yuan , Y. Zhang , K.‐X. Wang , et al., “Tuning the Energetic Performance of CL‐20 by Surface Modification Using Tannic Acid and Energetic Coordination Polymers,” ACS Omega 7, no. 12 (2022): 10469–10475, 10.1021/acsomega.1c07282. 35382280 PMC8973032

[50] J. Wang , Y. Yao , C. Zhang , et al., “Superstructured Macroporous Carbon Rods Composed of Defective Graphitic Nanosheets for Efficient Oxygen Reduction Reaction,” Advanced Science 8, no. 18 (2021): 2100120. 34323391 10.1002/advs.202100120 PMC8456237

[51] H. Li , R. Abbasi , Y. Wang , et al., “Liquid Metal‐Supported Synthesis of Cupric Oxide,” Journal of Materials Chemistry C 8, no. 5 (2020): 1656–1665, 10.1039/C9TC06883J.

[52] M. F. Sanad , A. R. Puente Santiago , S. A. Tolba , et al., “Co‐Cu Bimetallic Metal Organic Framework Catalyst Outperforms the Pt/C Benchmark for Oxygen Reduction,” Journal of the American Chemical Society 143, no. 10 (2021): 4064–4073. 33661615 10.1021/jacs.1c01096

[53] X. Zhang , X. Chao , N. Fei , et al., “Engineering the Grain Boundary and Surface Sites of Binary Cu–Mn Catalysts to Boost CO Oxidation,” Reaction Chemistry & Engineering 9, no. 10 (2024): 2659–2668, 10.1039/D4RE00222A.

[54] Y. Ye , J. Qian , H. Yang , et al., “Synergy Between a Silver–copper Surface Alloy Composition and Carbon Dioxide Adsorption and Activation,” ACS Applied Materials & Interfaces 12, no. 22 (2020): 25374–25382, 10.1021/acsami.0c02057. 32383583

[55] T. Wang , Z. Shi , F. Wang , et al., “Advanced Bifunctional Catalyst Design for Rechargeable Zinc–Air Batteries,” EES Catalysis 2, no. 3 (2024): 696–726, 10.1039/D4EY00014E.

[56] X. Shen , W. Liu , X. Gao , Z. Lu , X. Wu , and X. Gao , “Mechanisms of Oxidase and Superoxide Dismutation‐Like Activities of Gold, Silver, Platinum, and Palladium, and Their Alloys: A General Way to the Activation of Molecular Oxygen,” Journal of the American Chemical Society 137, no. 50 (2015): 15882–15891, 10.1021/jacs.5b10346. 26642084

[57] Y. Xu , Z. Zhou , N. Deng , et al., “Molecular Insights of Nanozymes From Design to Catalytic Mechanism,” Science China Chemistry 66, no. 5 (2023): 1318–1335, 10.1007/s11426-022-1529-y. 36817323 PMC9923663

[58] S. Li , H. Chen , Y. Qiu , C. Cui , W. Zhong , and J. Jiang , “Achieving the Selectivity of the Oxygen Reduction Reaction by Regulating Electron Spin States and Active Centers on Fe–Mn–N_6_–C Dual‐Atom Catalysts,” Journal of Materials Chemistry A 12, no. 47 (2024): 32855–32870, 10.1039/D4TA06650B.

[59] Q. Jia , N. Ramaswamy , U. Tylus , et al., “Spectroscopic Insights Into the Nature of Active Sites in Iron–Nitrogen–Carbon Electrocatalysts for Oxygen Reduction in Acid,” Nano Energy 29 (2016): 65–82, 10.1016/j.nanoen.2016.03.025.

[60] X. Kang , Y. Ren , J. Wang , et al., “A Noble Metal‐Enhanced Au@ CuO Heterostructure With Multienzyme‐Mimicking Activities for Colorimetric Detection of Tannic Acid,” The Analyst 149, no. 19 (2024): 4889–4898, 10.1039/D4AN01013B. 39171410

[61] S. Liang , T. Xin , and C. Wang , “Nanozymes in the Treatment of Diseases Caused by Excessive Reactive Oxygen Specie,” Journal of Inflammation Research 15 (2022): 6307–6328, 10.2147/JIR.S383239. 36411826 PMC9675353

[62] D. Aguilà , Y. Prado , E. S. Koumousi , C. Mathonière , and R. Clérac , “Switchable Fe/Co Prussian Blue Networks and Molecular Analogues,” Chemical Society Reviews 45, no. 1 (2016): 203–224, 10.1039/C5CS00321K. 26553752

[63] Y. Huang , L. Jia , S. Zhang , L. Yan , and L. Li , “Bimetallic Doped Carbon Dot Nanozymes for Enhanced Sonodynamic and Nanocatalytic Therapy,” Journal of Materials Chemistry B 13, no. 2 (2025): 588–598, 10.1039/D4TB01916D. 39575676

[64] E. O. Gubernatorova , X. Liu , A. Othman , et al., “Europium‐Doped Cerium Oxide Nanoparticles Limit Reactive Oxygen Species Formation and Ameliorate Intestinal Ischemia–Reperfusion Injury,” Advanced Healthcare Materials 6, no. 14 (2017), 10.1002/adhm.201700176. 28481012

[65] J. Cui , Q. Zhai , Z. Tan , et al., “Resveratrol‒Loaded Self‒Assembled Tetrahedral Framework Nucleic Acids Reshape the Epileptic Microenvironment by Regulating Oxidative Stress and Neuroinflammation via the SIRT_3_/SOD_2_ Pathway,” Journal of Nanobiotechnology (2026), 10.1186/s12951-026-04145-3. PMC13032418 41731565

[66] F.‐L. Zhang , Z. Hu , Y.‐F. Wang , et al., “Organoids Transplantation Attenuates Intestinal Ischemia/Reperfusion Injury in Mice Through L‐Malic Acid‐Mediated M_2_ Macrophage Polarization,” Nature Communications 14, no. 1 (2023): 6779, 10.1038/s41467-023-42502-0. PMC10600233 37880227

[67] H. J. Monkhorst and J. D. Pack , “Special Points for Brillouin‐Zone Integrations,” Physical Review B 13 (1976): 5188–5192, 10.1103/PhysRevB.13.5188.

[68] E. Louis , K. Paridaens , S. Al Awadhi , et al., “Modelling the Benefits of an Optimised Treatment Strategy for 5‐ASA in Mild‐to‐Moderate Ulcerative Colitis,” BMJ Open Gastroenterology 9, no. 1 (2022): 000853, 10.1136/bmjgast-2021-000853. PMC8845184 35165124

