# Supplementary material for: Tailored Porous Bimetallic Nanozyme Platform for Full‐Cycle Therapeutics of Intestinal Ischemia/Reperfusion
Source: Adv Sci (Weinh). 2026 Mar 31;13(34):e20748. doi: 10.1002/advs.202520748 (PMC13285119; doi:10.1002/advs.202520748)
Supplement: Supplementary file 1 — Supporting File: advs75059‐sup‐0001‐SuppMat.docx. [file ADVS-13-e20748-s001.docx]

**Supplementary Information**

**Tailored Porous Bimetallic Nanozyme Platform for Full-cycle Therapeutics of Intestinal Ischemia/reperfusion**

Chenghao Qiu, Wenjing Xian, Mengxue Wang, Guozhi Zhao, Yanhuo Zhang, Zhiyu Zhou, Bo Sheng, Zhuo Zhen, Xie Liu, Shouwen Diao, Mengwei Tu, Chen Qiu, Huixian Wang, Jianbo Zhang^*^, Peng Zhu^*^, and Tong Li^*^

C.H. Qiu, M. Wang, Y. Zhang, B. Sheng, Z. Zhen, X. Liu, S. Diao, M. Tu, J. Zhang, P. Zhu, T. Li

Department of Gastrointestinal Surgery, The Second Affiliated Hospital of Chongqing Medical University, Chongqing 400016, P. R. China

E-mail：tonglicqmu@hospital.cqmu.edu.cn；zupeng@cqmu.edu.cn；zhangjianbo@hospital.cqmu.edu.cn

G. Zhao

Department of Urology Surgery, The First Affiliated Hospital of Chongqing Medical University, Chongqing 400016, P. R. China

W. Xian

Department of Anesthesiology, The First Affiliated Hospital of Chongqing Medical University, Chongqing 400016, P. R. China

Z. Zhou

Department of Respiratory Medicine, The Second Affiliated Hospital of Chongqing Medical University, Chongqing 400016, P. R. China

C. Qiu

Department of Rehabilitation Medicine, The Second Affiliated Hospital of Chongqing Medical University, Chongqing 400016, P. R. China

H. Wang

Department of Oncology, Laboratory of Immunity, Inflammation & Cancer, The First Affiliated Hospital of Chongqing Medical University, Chongqing, 400016, Chin

**Supplementary Table**

| Material | BET Surface Area (m²/g) | Total Pore Volume (cm³/g) | Average Pore Size (BJH, nm) |
| --- | --- | --- | --- |
| PB | 350.46 | 0.164 | 2.18 |
| MPB@TA | 344.08 | 0.169 | 3.06 |
| MPB@TA-Cu | 348.46 | 0.175 | 3.14 |

**Tbale S1.** Quantitative summary of the specific surface area, pore volume, and average pore size for PB, MPB@TA, and MPB@TA-Cu.

**Supplementary Figures**


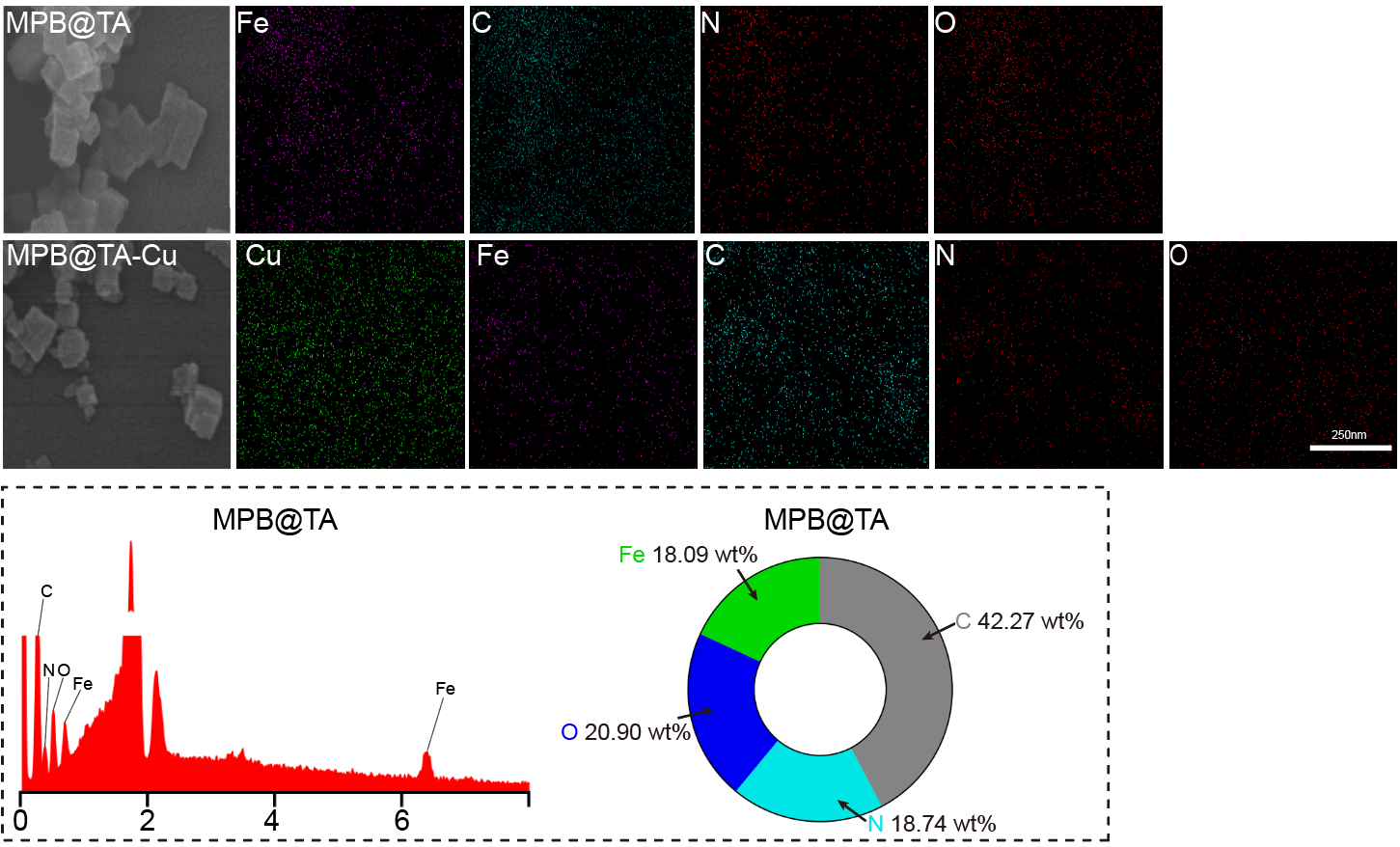


**Figure S1.** Presents scanning electron microscope (SEM) images, element distribution maps, element percentage charts, and corresponding total spectra for MPB@TA and MPB@TA-Cu materials, thereby revealing their morphology, elemental composition, and distribution. Scale bars: 250 nm.


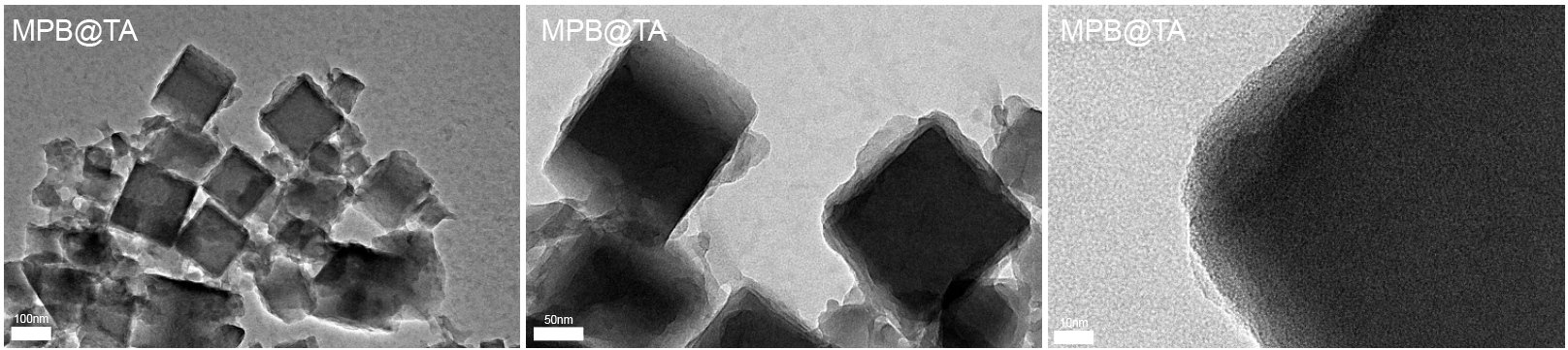


**Figure S2.** Presents transmission electron microscope (TEM) images of MPB@TA material at various magnifications, with scale bars indicating 100 nm, 50 nm, and 10 nm from left to right.


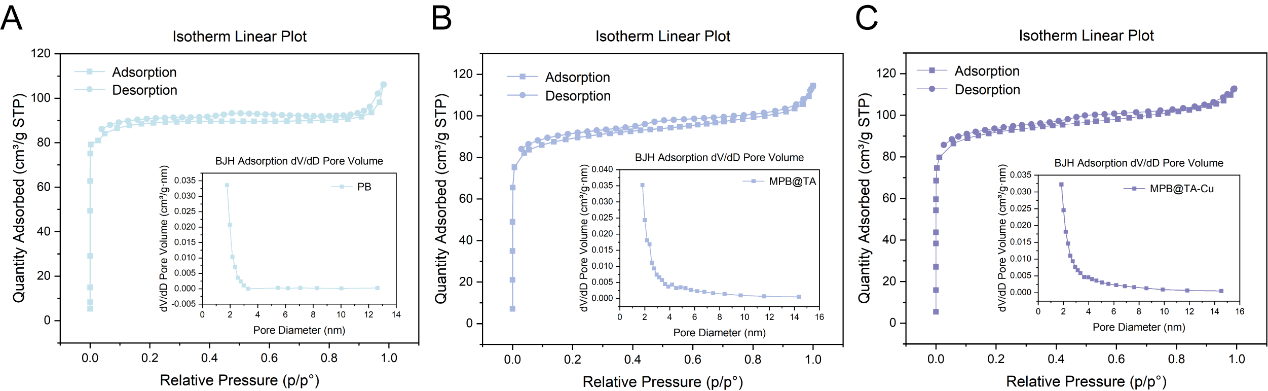


**Figure S3.** Nitrogen (N₂) adsorption-desorption isotherms and corresponding pore size distributions of the synthesized nanoparticles. (A-C) Isotherm linear plots of (A) PB, (B) MPB@TA, and (C) MPB@TA-Cu. The insets display the corresponding Barrett-Joyner-Halenda (BJH) pore size distribution curves calculated from the adsorption branches.


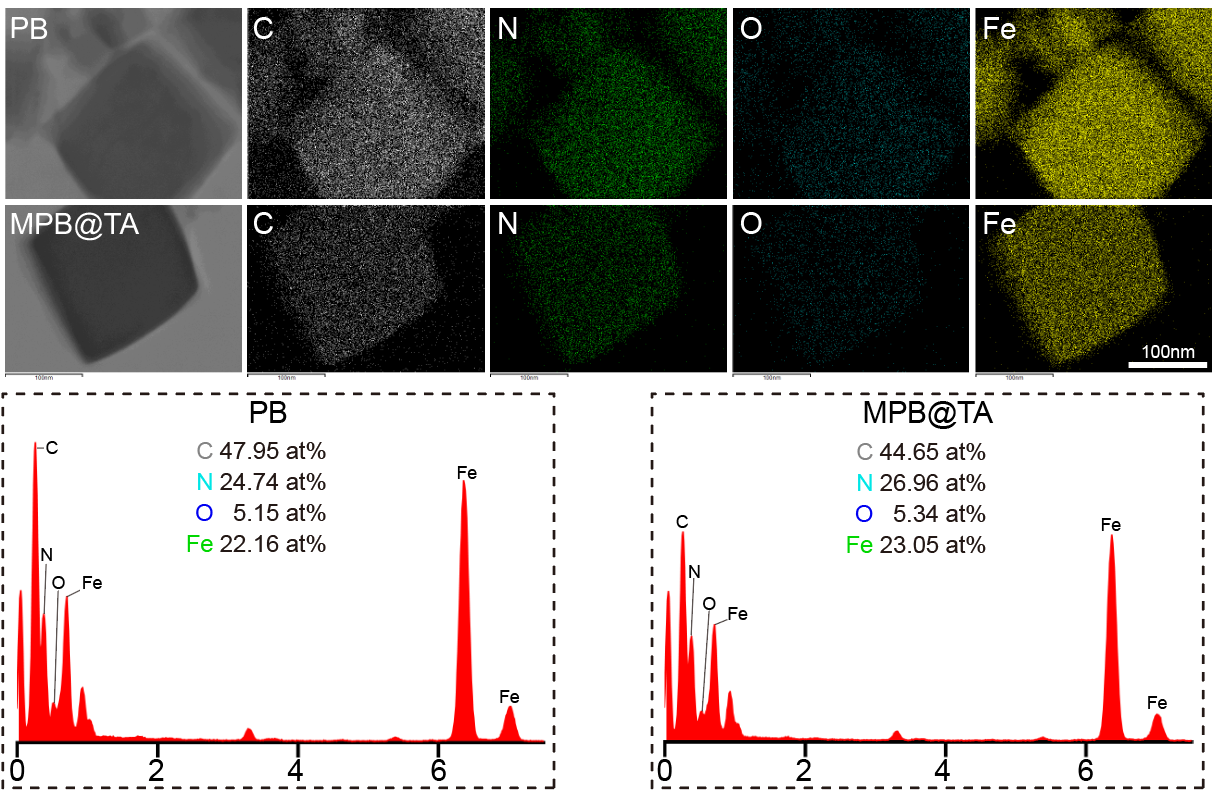


**Figure S4.** TEM images and energy-dispersive X-ray spectroscopy (EDS) mappings of iron (Fe), carbon (C), and nitrogen (N) elements in PB and MPB@TA materials, accompanied by the corresponding total spectrum. Scale bars: 100 nm.


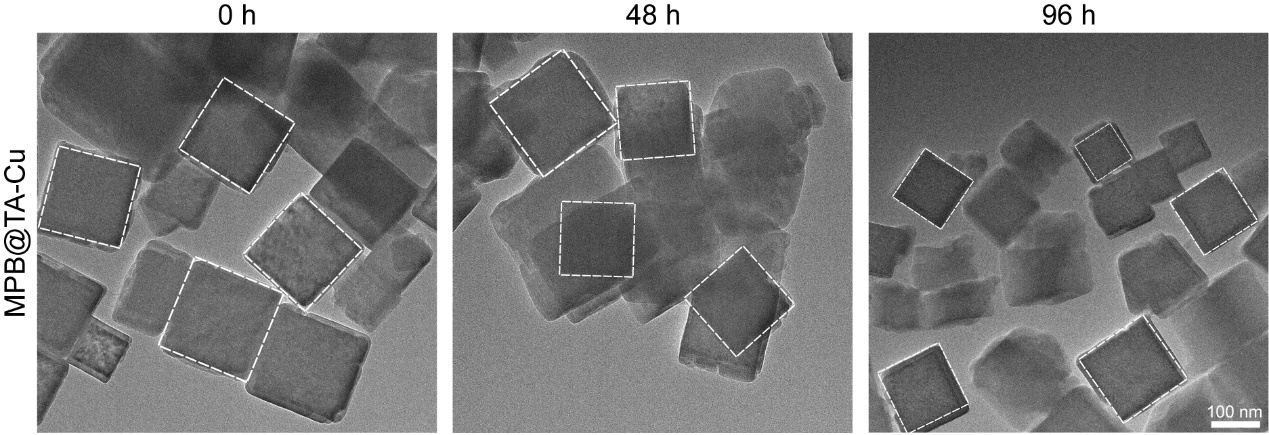


**Figure S5.** SEM images of MPB@TA-Cu-Ma nanoparticles after incubation in physiological buffer for 0 h (A), 48 h (B), and 96 h (C). Scale bars: 100 nm.


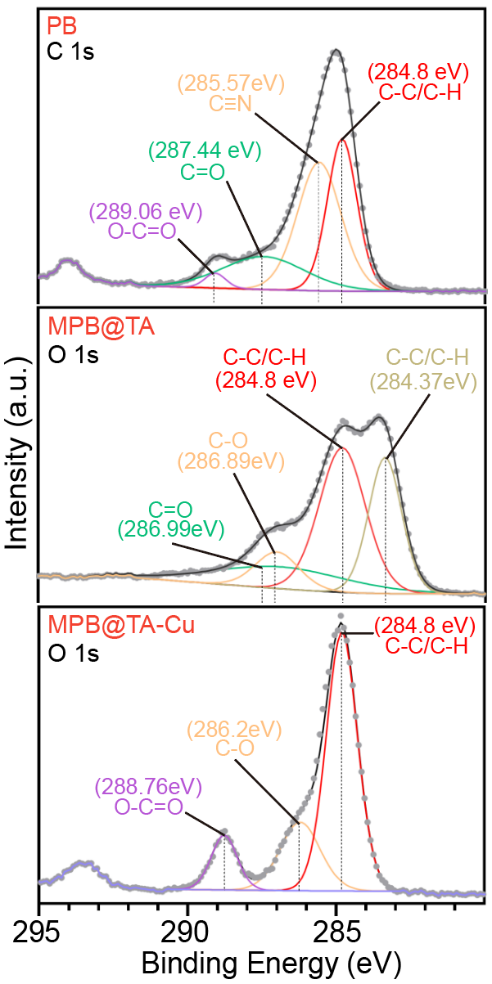


**Figure S6.** X-ray photoelectron spectroscopy (XPS) analysis of PB, MPB@TA, and MPB@TA-Cu materials, showing the comparison of C 1s chemical bond composition and intensity differences.


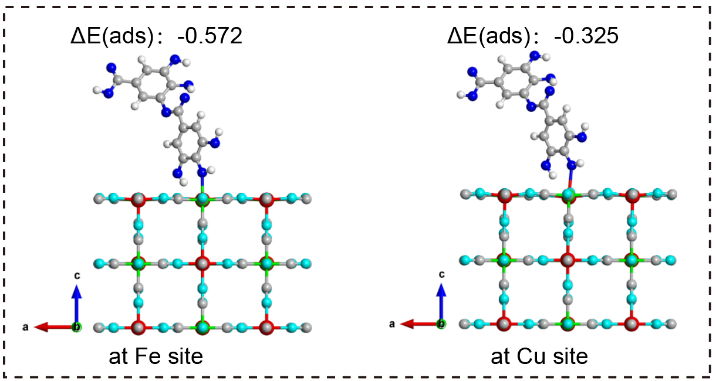


**Figure S7.** Density Functional Theory (DFT)-optimized structural models of tannic acid (TA) adsorbed on the Fe site and Cu site, together with the corresponding adsorption energies.


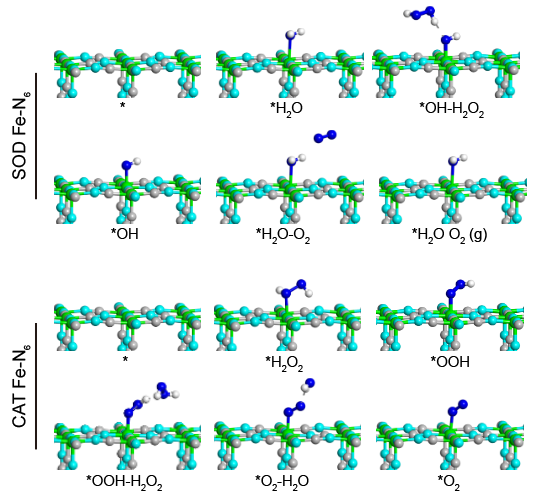


**Figure S8.** Computational surface models of FePB (200) featuring Fe- N₆ sites as the active centers, along with the atomic conformational changes occurring during SOD-mimic, and CAT-mimic catalytic processes.


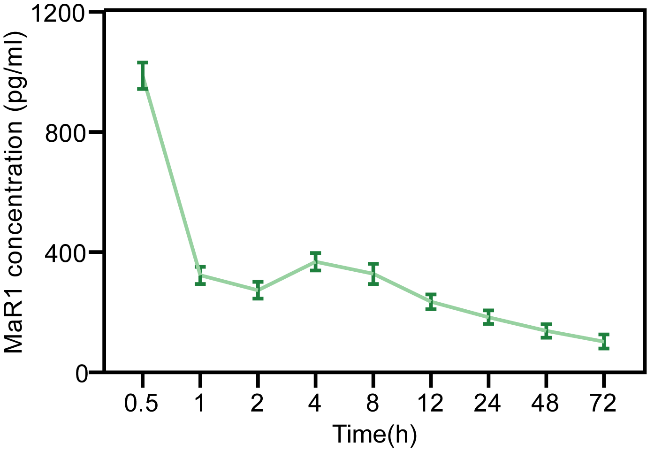


**Figure S9.** **Release profile of MaR1 from MPB@TA-Cu-Ma in PBS buffer.** Absolute concentration of MaR1 released at each designated time interval over 72 h. Data are presented as mean ± SEM (n = 4).


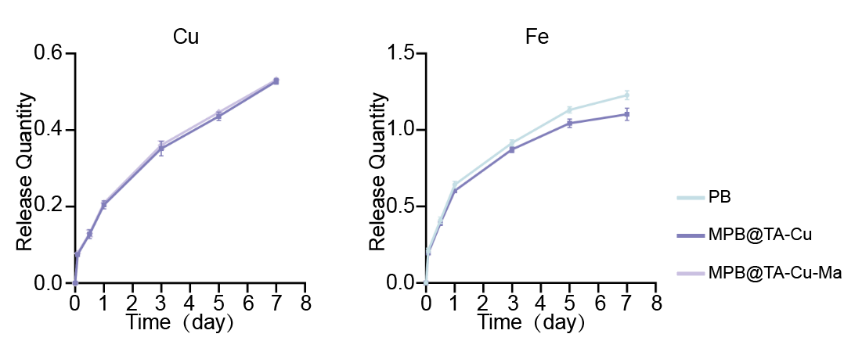


**Figure S10.** Release profiles of copper (Cu) and iron (Fe) from different treatment groups over time. The release quantities of Cu and Fe were measured using inductively coupled plasma (ICP) detection. The treatment groups include PB, MPB@TA-Cu, and MPB@TA-Cu-Ma. Data are expressed as the mean ± standard error of the mean (SEM) from three independent replicates per group.


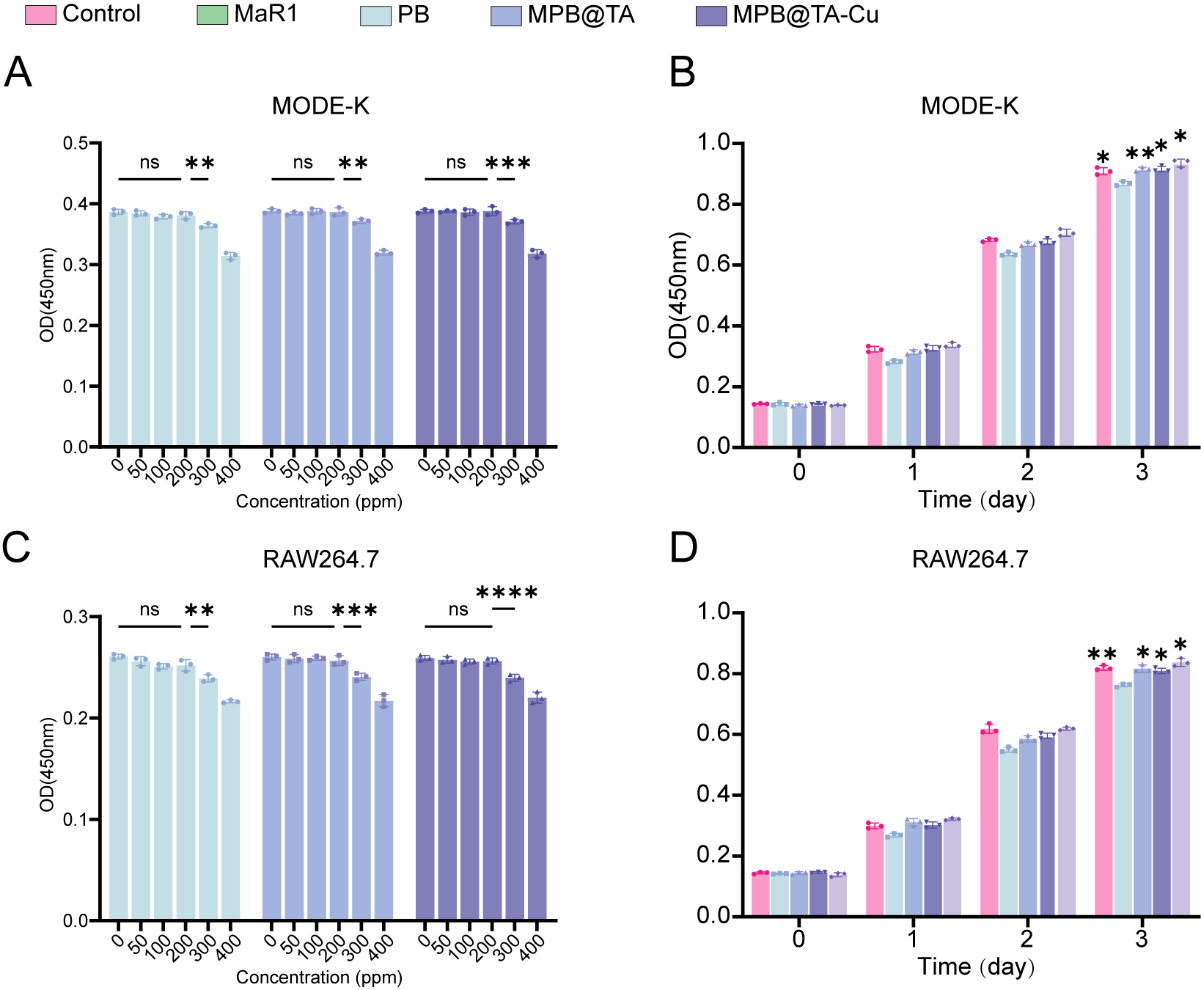


**Figure S11.** **In vitro biocompatibility and proliferation profiles of MODE-K and RAW264.7 cells.** (A, C) Concentration-dependent cytotoxicity in MODE-K intestinal epithelial cells (A) and RAW264.7 macrophages (C) treated with the indicated formulations (0–400 ppm). (B, D) Time-dependent cell proliferation of MODE-K cells (B) and RAW264.7 macrophages (D) over a 3-day culture period. Cell viability was evaluated via CCK-8 assay. Data are presented as mean ± SEM (n = 3). Statistical significance was determined by two-way ANOVA followed by Tukey's post hoc test. *p < 0.05, **p < 0.01, ***p < 0.001, ****p < 0.0001; ns, not significant.


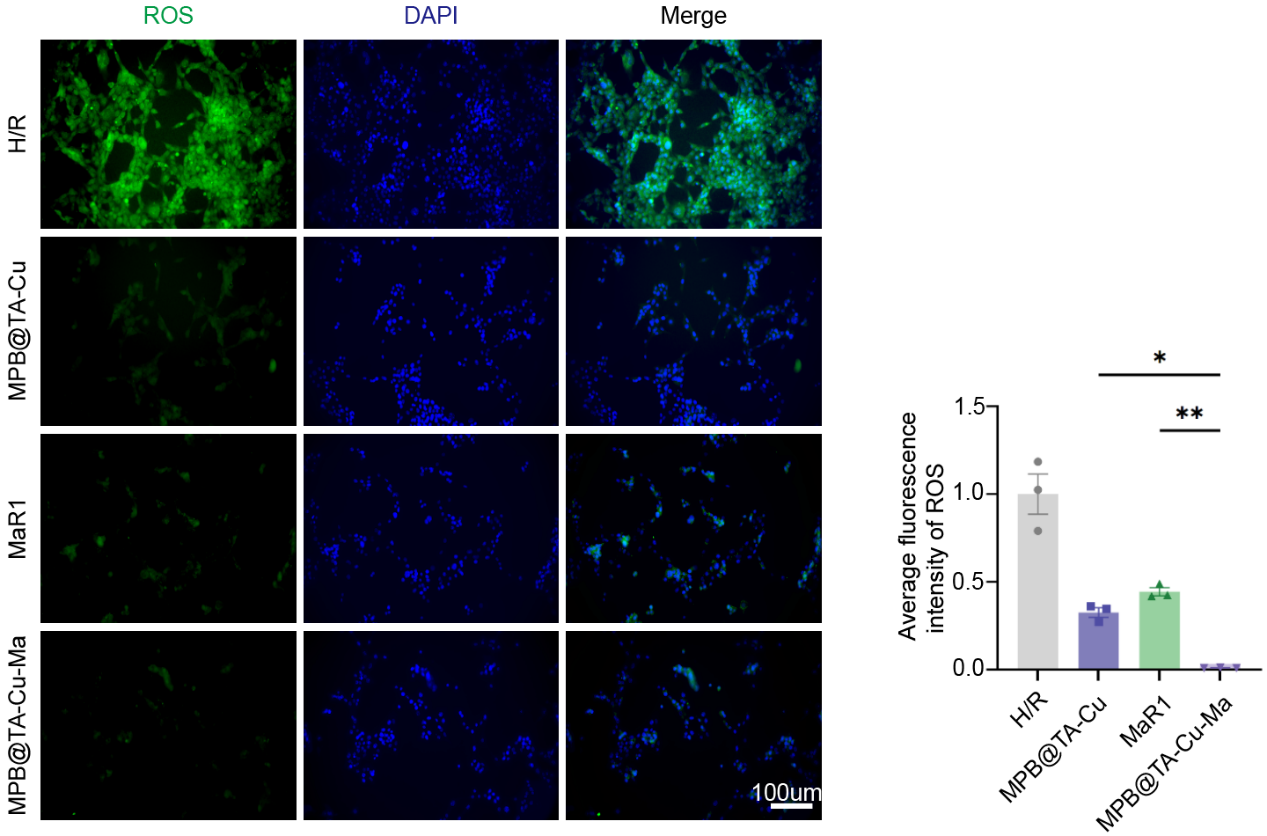


**Figure S12.** MODE-K cells pretreated with MPB@TA-Cu, Maresin-1, or MPB@TA-Cu-Ma were subjected to H/R injury. Intracellular ROS levels were detected using fluorescence microscopy. Green: ROS; Blue: DAPI (nuclei). Scale bar = 100 μm. Data are presented as mean ± SEM (n = 3 biologically independent experiments). Statistical significance was determined by one-way ANOVA followed by Tukey's post hoc test. *p < 0.05, **p < 0.01.


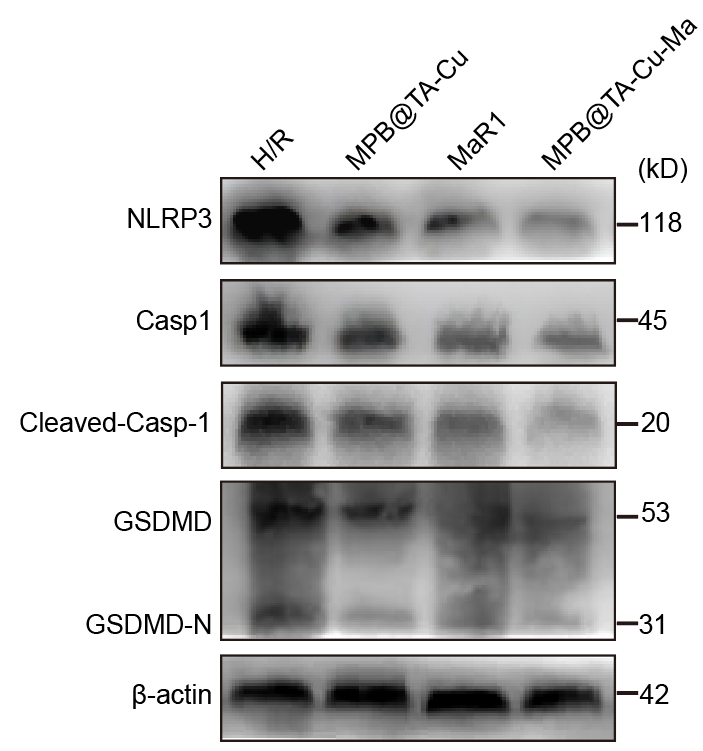


**Figure S13.** RAW264.7 cells pretreated with MPB@TA-Cu, Maresin-1, or MPB@TA-Cu-Ma were subjected to H/R injury, and the expression levels of NLRP3,Caspase1,Cleaved-caspase1,GSDMD and its cleaved form (GSDMD-N) were assessed by Western blot analysis.


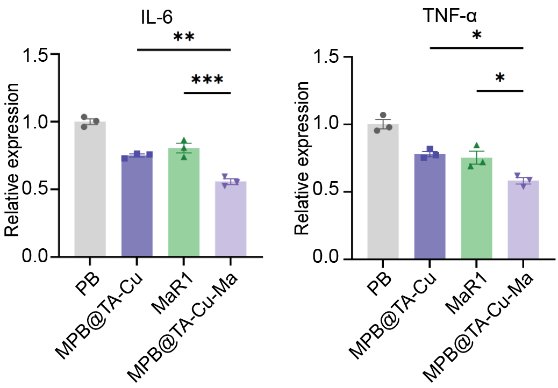


**Figure S14.** **ELISA quantification of IL-6 and TNF-**$\alpha$ **in RAW264.7 supernatants.** Relative expression levels of pro-inflammatory cytokines IL-6 (left) and TNF-$\alpha$ (right) in RAW264.7 macrophages treated with PB, MPB@TA-Cu, MaR1, or MPB@TA-Cu-Ma. Data are presented as mean ± SEM (n = 3 biologically independent experiments). Statistical significance was determined by one-way ANOVA followed by Tukey's post hoc test. *p < 0.05, **p < 0.01, ***p < 0.001.


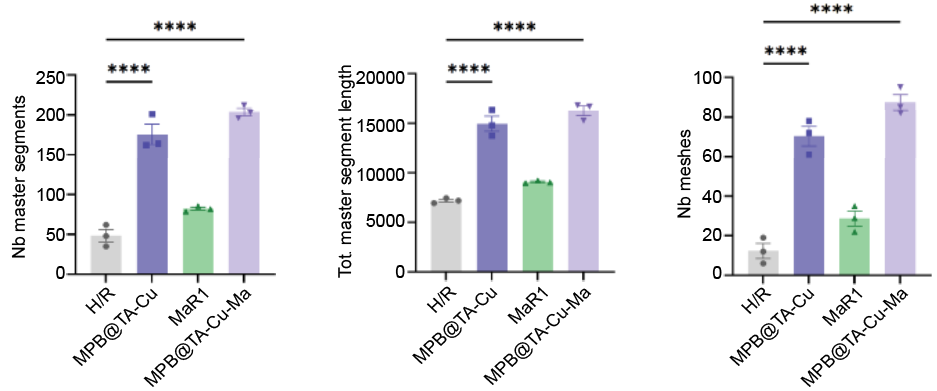


**Figure S15.** **Quantitative assessment of angiogenesis in HUVECs.** Quantitative analysis of the number of master segments (left), total master segment length (middle), and number of meshes (right) in human umbilical vein endothelial cells (HUVECs) subjected to hypoxia/reoxygenation (H/R) injury and subsequently treated with MPB@TA-Cu, MaR1, or MPB@TA-Cu-Ma. Data are presented as mean ± SEM (n = 3 biologically independent experiments). Statistical significance was determined by one-way ANOVA followed by Tukey's post hoc test. ****p < 0.0001.


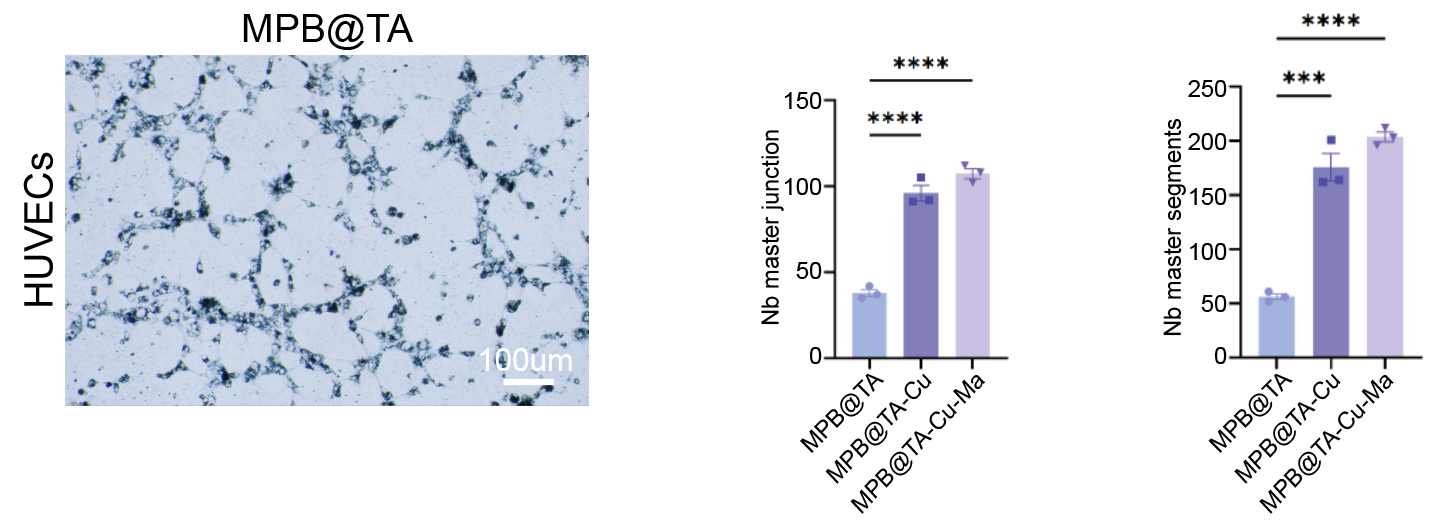


**Figure S16.** **Representative microscopic image and quantitative assessment of angiogenesis in HUVECs**. Representative bright-field image of human umbilical vein endothelial cells (HUVECs) treated with MPB@TA (left). (Representative images for the other treatment groups are shown in Figure 4L.) Quantitative analysis of the number of master junctions (middle) and number of master segments (right) in HUVECs following treatment with MPB@TA, MPB@TA-Cu, or MPB@TA-Cu-Ma. Scale bar = 100 μm. Data are presented as mean ± SEM (n = 3 biologically independent experiments). Statistical significance was determined by one-way ANOVA followed by Tukey's post hoc test. ***p < 0.001, ****p < 0.0001.


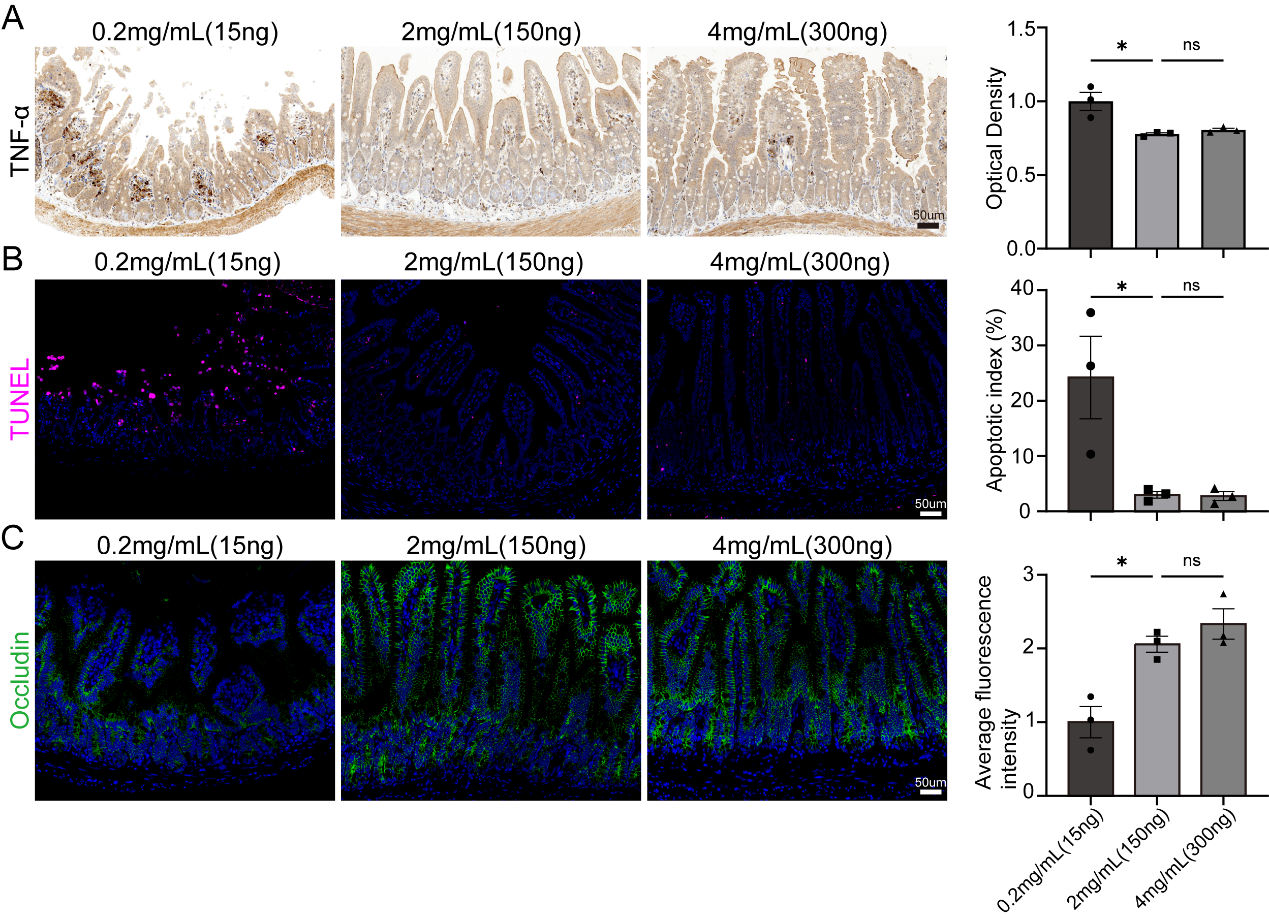


**Figure S17.** **Dose-dependent therapeutic effects of MPB@TA-Cu-Ma in the intestinal I/R injury model.** (A) Representative immunohistochemistry images and quantification of TNF-α optical density at 6 h post-reperfusion. (B) Representative TUNEL staining images and quantification of the apoptotic index at 6 h post-reperfusion. Magenta: TUNEL-positive cells; Blue: nuclei. (C) Representative immunofluorescence images and average fluorescence intensity analysis of the tight junction protein Occludin at 96 h post-reperfusion. Green: Occludin; Blue: nuclei. Mice were intraperitoneally treated with MPB@TA-Cu-Ma at 0.2, 2, or 4 mg/mL (100 μL per mouse, containing 15, 150, and 300 ng MaR1, respectively). Scale bar = 50 μm. Data are presented as mean ± SEM (n = 3 biologically independent animals). Statistical significance was determined by one-way ANOVA followed by Tukey's post hoc test. *p < 0.05; ns, not significant.


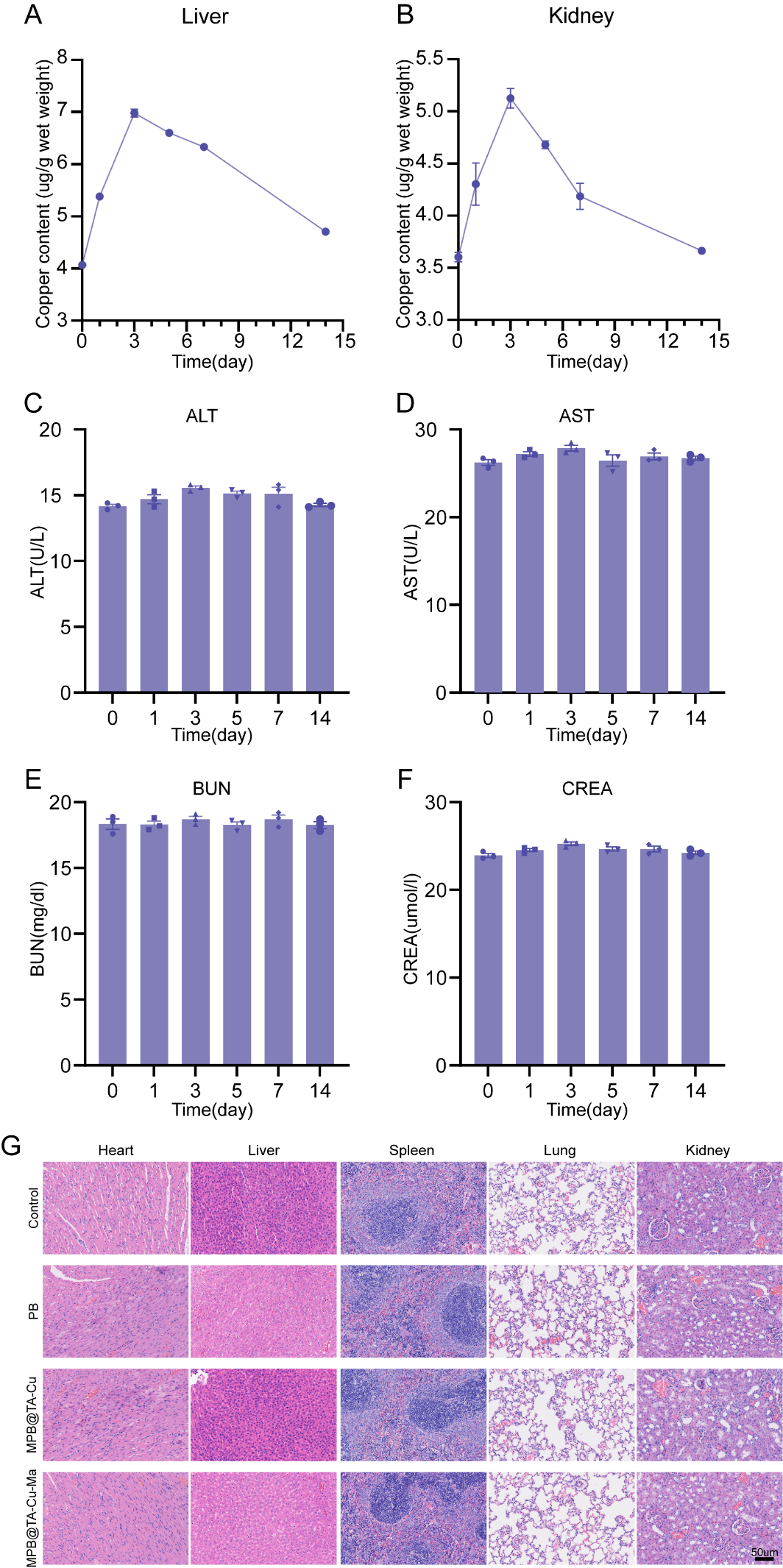


**Figure S18.** **In vivo biosafety assessment of MPB@TA-Cu and related nano-formulations.** (A, B) Time-dependent copper content in the liver (A) and kidney (B) over a 14-day period. (C–F) Blood biochemistry analysis of liver function markers, ALT (C) and AST (D), and kidney function markers, BUN (E) and CREA (F), at designated time points over 14 days. (G) Representative H&E staining images of major organs (heart, liver, spleen, lung, and kidney) following treatment with Control, PB, MPB@TA-Cu, or MPB@TA-Cu-Ma. Scale bar = 50 μm. Data are presented as mean ± SEM (n = 3 biologically independent animals). Statistical significance for (C–F) was determined by one-way ANOVA followed by Tukey's post hoc test; no significant differences were observed across the evaluated time points.


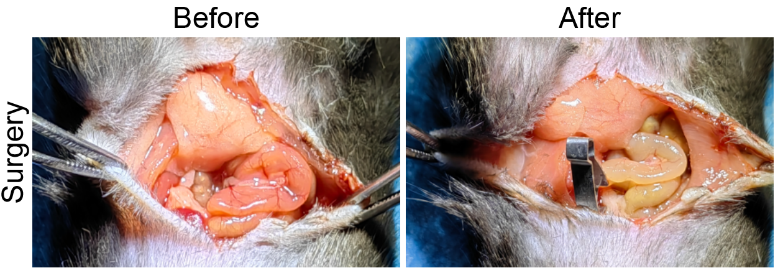


**Figure S19.** **Representative surgical images of the small intestinal I/R model in C57BL/6 mice.** Representative macroscopic images of the exposed small intestine before (left) and after (right) the induction of ischemia. The right panel clearly displays the application of an atraumatic microvascular clip to the superior mesenteric artery (SMA), resulting in obvious pallor of the intestinal tissue, which visually confirms successful ischemic occlusion.


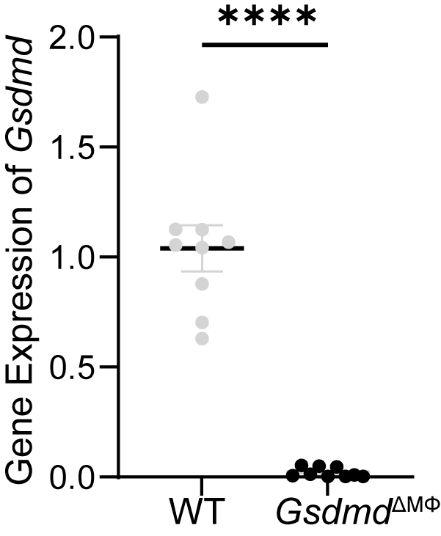


**Figure S20. Gene expression of *Gsdmd* in macrophages isolated from WT and *Gsdmd*^ΔMΦ^ mice**. Relative mRNA expression levels of *Gsdmd* in primary macrophages isolated from wild-type (WT) mice and macrophage-specific *Gsdmd* knockout (*Gsdmd*^ΔMΦ^) mice, as determined by quantitative real-time PCR Data are presented as mean ± SEM (n = 8 biologically independent samples). Statistical significance was determined by an unpaired two-tailed Student's t-test. ****P < 0.0001.


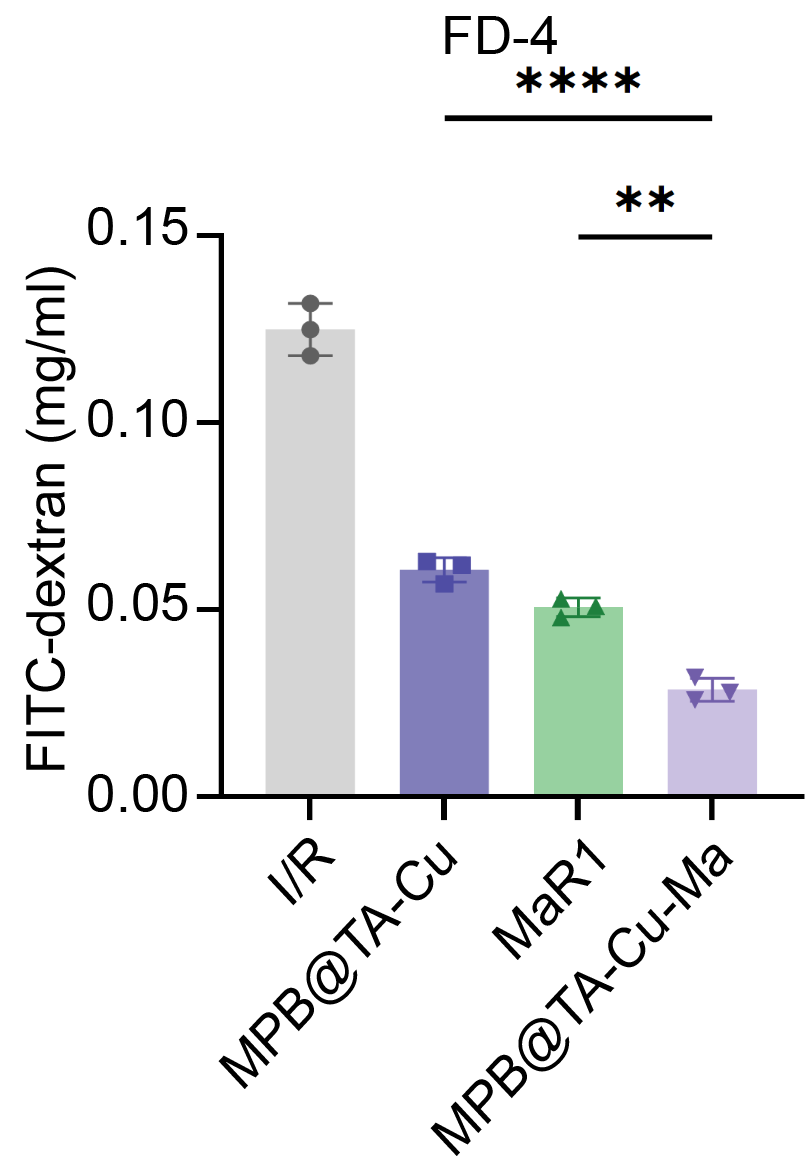


**Figure S21. Assessment of intestinal permeability in the I/R injury model**. Serum concentrations of FITC-dextran (FD-4) in C57BL/6 mice subjected to intestinal I/R injury following pretreatment with MPB@TA-Cu, MaR1, or MPB@TA-Cu-Ma. FITC-dextran was administered via gastric gavage, and serum fluorescence was subsequently evaluated to assess intestinal barrier integrity. Data are presented as mean ± SEM (n = 3 biologically independent animals). Statistical significance was determined by one-way ANOVA followed by Tukey's post hoc test. ** p < 0.01, **** p < 0.0001.


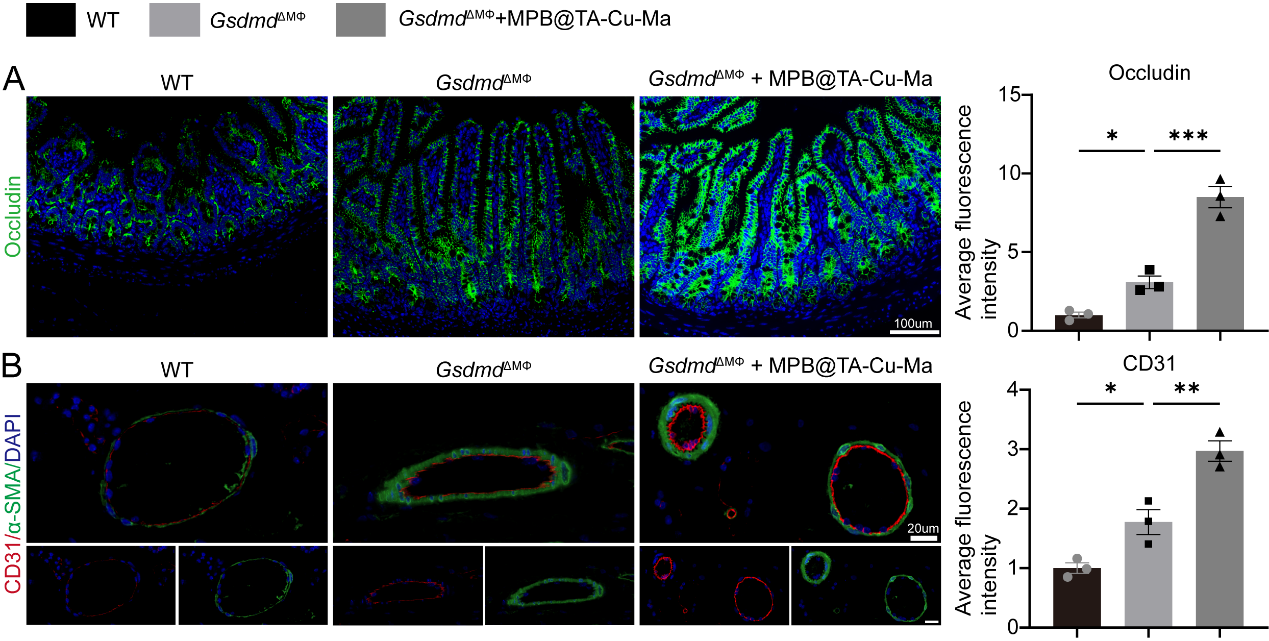


**Figure S22.** **Non-pyroptosis-dependent regenerative effects of MPB@TA-Cu-Ma during the recovery phase of intestinal I/R injury**. (A) Immunofluorescence analysis of the tight junction protein Occludin in intestinal tissues from WT, *Gsdmd*^ΔMΦ^, and MPB@TA-Cu-Ma-treated *Gsdmd*^ΔMΦ^ mice at 96 h post-reperfusion, with corresponding quantitative bar graphs showing individual data points. (B) Immunofluorescence analysis of the vascular endothelial marker CD31 (red) and vascular smooth muscle marker α-SMA (green) in intestinal tissues from the indicated groups at 96 h post-reperfusion, with corresponding quantitative bar graphs showing individual data points. Scale bars: 100 µm (A), 20 µm (B). Data are presented as mean ± SEM (n = 3 biologically independent mice). Statistical significance was determined by one-way ANOVA followed by Tukey's post hoc test. *p < 0.05, **p < 0.01, ***p < 0.001.


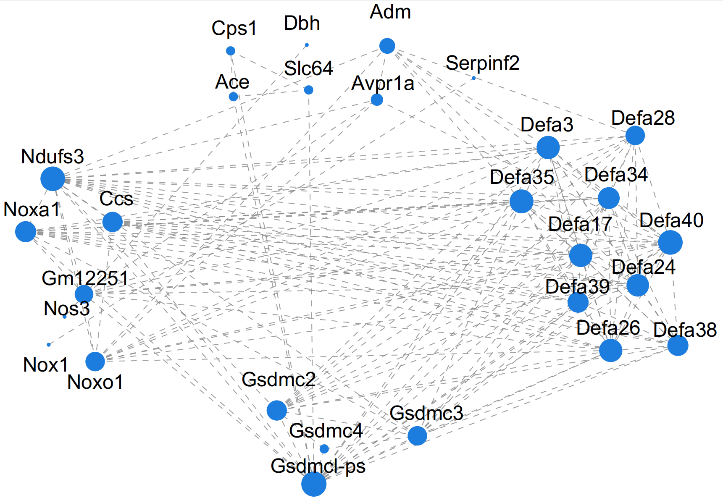


**Figure S23.** **Interaction network of differential genes.** The network illustrates the predicted functional interactions or co-expression correlations among key DEGs. The circular nodes represent individual genes (highlighting distinct clusters such as the *Defa* family, *Gsdmc* family, and Nox-related genes), while the dashed lines (edges) indicate molecular interactions. The size of each node is proportional to its degree of connectivity within the network, highlighting potential hub genes (e.g., *Ndufs3*, *Adm*, and *Gsdmcl-ps*).
